# QTL identified that influence tuber length–width ratio, degree of flatness, tuber size, and specific gravity in a russet-skinned, tetraploid mapping population

**DOI:** 10.3389/fpls.2024.1343632

**Published:** 2024-03-22

**Authors:** Jaebum Park, Jonathan Whitworth, Richard G. Novy

**Affiliations:** Small Grains and Potato Germplasm Research Station, United States Department of Agriculture—Agricultural Research Service, Aberdeen, ID, United States

**Keywords:** potato tuber shape visual assessment, tuber length-width ratio, tuber width-depth ratio, specific gravity, tuber size, tetraploid potato QTL analysis

## Abstract

Potato tuber shape, size, and specific gravity are important agronomic traits in the russet market class of potatoes with an impact on quality, consistency, and product recovery of processed foods such as French fries. Therefore, identifying genetic regions associated with the three traits through quantitative trait locus/loci (QTL) analysis is a crucial process in the subsequent development of marker-assisted selection for use in potato breeding programs. QTL analysis was conducted on a tetraploid mapping population consisting of 190 individuals derived from the cross between two russet-skinned parents, Palisade Russet and the breeding clone ND028673B-2Russ. Field data collected over a 2-year period and used in the QTL analyses included tuber length–width and width–depth ratios that were obtained using a digital caliper. The width–depth ratio provided an assessment of the “flatness” of a tuber, which is of importance in potato processing. To cross-validate the accuracy and differences among tuber shape measurement methods, a trained evaluator also assessed the identical tubers based on 1–5 scale (compressed to long) visual assessment method. Furthermore, the weights of analyzed tubers and specific gravities were also collected during the phenotyping process for each mapping clone. A major tuber shape QTL was consistently observed on chromosome 10 with both the length–width ratio and visual assessments. On chromosome 4, a significant QTL for tuber shape from the visual assessment phenotypic data was also detected. Additionally, a tuber shape-related QTL on chromosome 6 was also detected from the length–width ratio data from 2020. Chromosome 2 was also identified as having a significant QTL for the width–depth ratio, which is of importance in influencing the flatness of a tuber. One significant QTL for tuber weight (i.e., tuber size) was observed on chromosome 5, and a significant QTL for specific gravity was found on chromosome 3. These significant and major QTL should be useful for developing marker-assisted selection for more efficient potato breeding.

## Introduction

Breeding superior potato cultivars for processed products is important to US potato breeding programs with over 65% of the total US potato production (~19.2 million metric tons in 2019) used for producing processed products, including French fries, chips, and refrigerated and frozen items utilized by food services (USDA, 2020). Two tuber traits, tuber shape and specific gravity, are of importance in potato processing. For instance, round shape tubers are preferred for making potato chips, while long tuber shapes, typical of the russet market class, are preferred for French fry production; deviations from these preferred shapes can significantly increase the waste ratio during processing (Vreugdenhil et al., 2011; Chen et al., 2019). The specific gravity, which primarily represents tuber starch content, directly affects the amount of oil required for processing and the textural quality of the final product (Stark et al., 2020). Even with the high popularity of processed foods and the important roles of the two traits in processed potato production, quantitative trait locus/loci (QTL) analyses for tuber shape and specific gravity in tetraploid potatoes have been conducted relatively rarely compared to potato pathogen and pest resistances. Therefore, the purpose of this study was to identify QTL influencing tuber shape and specific gravity in a tetraploid mapping population that could subsequently be used in developing marker-assisted selection (MAS) for these important processing traits.

Park et al. (2021) performed QTL analysis with a biparental tetraploid mapping population derived from two russet potato cultivars (Rio Grande Russet and Premier Russet). The most impactful QTL on tuber length was consistently detected on chromosome 10 based on a 2-year phenotypic data, with additional minor QTL found on chromosomes 4 and 7 (Park et al., 2021). Through extensive reference research and comparative study, the significant QTL on chromosome 10 seemed to be the major tuber shape-controlling gene (or locus), Ro (Masson, 1985), and had been repeatedly localized at a similar position across multiple populations having different genetic backgrounds and ploidy levels (Sharma et al., 2013; Hirsch et al., 2014; Endelman and Jansky, 2016; Hara-Skrzypiec et al., 2018; Spud Database, 2020; Park et al., 2021). Potato tuber shape can have a continuous distribution between round and long (Masson, 1985; De Jong and Burns, 1993; Hara-Skrzypiec et al., 2018). Diverse tuber shape-associated genes or QTL have been reported on chromosomes 2, 3, 4, 5, 6, 8, 9, 10, and 11; populations have represented diverse genetic backgrounds and have comprised full-sib diploid, F2, gynogenic dihaploid, and tetraploid populations (Śliwka et al., 2008; Bradshaw et al., 2008; Prashar et al., 2014; Hara-Skrzypiec et al., 2018; Manrique-Carpintero et al., 2018; Meijer et al., 2018). Despite the valuable findings mentioned above, some areas still need to be supplemented or cross-validated. For instance, the previously referenced research projects for tuber shape did not reflect a three-dimensional tuber shape; instead they rely on simpler two-dimensional information such as length–width ratio or visual assessment using ordinal scales (e.g., 1—compressed, 2—round, … 5—long) (Śliwka et al., 2008; Bradshaw et al., 2008; Prashar et al., 2014; Endelman and Jansky, 2016; Hara-Skrzypiec et al., 2018; Manrique-Carpintero et al., 2018; Meijer et al., 2018). The depth of a tuber (or flatness) can also impact potato processing and associated product recovery, with this tuber dimension not being recorded and analyzed for associated QTL in previous studies.

Furthermore, even though the two techniques, continuous numerical measurement (e.g., length–width ratio) and ordinal subjective scale (e.g., visual assessment based on the SolCAP tuber shape 1–5 scale), have been used selectively according to the preferences of researchers, a comparison of the two methodologies and associated QTL concordance has not been investigated to the best of our knowledge. This study compared these two differing methodologies for assessing tuber shape. In addition, tuber weights and specific gravities were also measured, and QTL analyses were conducted.

Results of this study were compared with the research results of Park et al. (2021) to identify whether the dissimilar russet parents of this study’s mapping population and Park’s previous mapping population impacted QTL for tuber shape and specific gravity, i.e., were QTL for these traits population specific, or was their concordance for QTL identified in these two divergent tetraploid mapping populations representing the russet market class?

## Materials and methods

### Plant material

In 2008, the hybridization between Palisade Russet (female parent, late blight resistant) with breeding clone ND028673B-2Russ (male parent, late blight susceptible) was conducted, in Aberdeen, ID, USA, to develop a biparental mapping population (Novy et al., 2012; Susie Thompson, North Dakota State University, personal communication regarding late blight susceptibility of the male parent). This mapping population was labeled “A08241” and originally used for QTL analysis for late blight, early blight, and *Verticillium* wilt resistance in a previous study (Park et al., 2023). In the current study, all 190 individuals of A08241 and their two parents were also used for linkage and QTL mapping for tuber shape, size, and specific gravity. The female parent, cv., Palisade Russet, has a long tuber length and high specific gravity, satisfying processed food purposes (e.g., French fries) as well as representing a russet potato. Additionally, it had late blight, *Verticillium* wilt, early blight resistances, and a low incidence of sugar ends (Novy et al., 2012). Such detailed information on ND028673B-2Russ is not available where it was not released as a potato cultivar; it, nonetheless, represented the russet market class. Russet Burbank, the most widely grown russet variety in North America, was added as a standard control during field experiments with the A08241 mapping population. However, Russet Burbank’s phenotypic data were omitted from QTL and data analyses because it was not a part of the A08241 mapping population.

### Measurements of tuber shape, specific gravity, and tuber weight (=tuber size)

The A08241 mapping population and their two parents were planted for this research project in May 2019 and 2020. The eight-hill plots of the mapping population were replicated twice in a field in a randomized complete block design in each of the 2 years. After growing for 145 and 147 days in 2019 and 2020, respectively (Park et al., 2023), 10 tubers of each plot were randomly selected during the harvesting seasons. In other words, 40 tubers from each clone were selected over the 2 years (=10 tubers from each plot × 2 replicated blocks in each year × 2 years). Exceptions were observed for four clones (A08241-83, -88, -104, and -177), where only 30 tubers could be obtained due to poor emergence or yields occurring in one replicate during their field experiments in one of the 2 years. The selected tubers were placed on a lab workbench; their length and width were measured using a digital caliper. The definition of the length was the longest straight line between stem end and bud end (**Supplementary Figure 1**). The width was defined as the widest line perpendicular to the length. The simple equation “length/width” was used to calculate the length–width ratio giving numerical and objective data. The depth was additionally assessed to achieve an idea of the thickness of each tuber. A tuber was horizontally rotated by 90°, and then, the broadest straight distance perpendicular to the width was recorded as the depth (**Supplementary Figure 1**). Another equation, “width/depth,” was used to obtain the width–depth ratio estimating whether a tuber is relatively flat or swollen. iGAGING® Ip54 digital caliper (Enjoy Accuracy® with iGAGING® Tools, San Clemente, CA, USA) was used for all the measurements above. The units used for the length, width, and depth were in inches with the tuber measurements then being used in the calculation of the aforementioned tuber ratios. Each clone was also assessed visually to probe any distinction between human judgment and numerical values in tuber shape interpretation. For the visual assessment, the same 10 tubers, whose three-dimensional values (length, width, and depth) were already measured using the digital caliper, were aligned on the lab workbench. Then, a trained evaluator assigned the best score reflecting the common appearance feature of the 10 tubers based on a scale developed originally by the NE1014 Multi-State Research Project (SolCAP, 2009). The evaluator assessed the totality of the 10 tubers in assigning the shape score with a rating of “1” (Compressed) to “5” (Long) (**Supplementary Figure 2**) (SolCAP, 2009). After completing the tuber shape evaluation processes, the specific gravity data for each plot was collected through “weight in air/(weight in air − weight in water).” Finally, the individual weights for each of the 10 tubers were measured initially in ounces (oz) and subsequently converted to grams (g). It should be noted that the tuber weight did not indicate the yield of each clone but represented the size information of each measured tuber in this study because only 10 tubers of each plot were randomly selected as a representative subsample of the plot.

### Best linear unbiased predictor analyses for tuber shape, weight, and specific gravity

The collected raw phenotype data were then investigated with the following mixed-effect models resulting in estimates of variance components and prediction of the genetic values for the genotypes (Fernando and Grossman, 1989; Barr et al., 2013; Peixouto et al., 2016):


(1) $$y_{ijlk}=\mu \;+\;G_{i}\;+\;B_{j(k)}\;+\;R_{l(k)}\;+\;Y_{k}+{\left(GY\right)}_{ik}+\;{\text{ε}}_{ijlk}$$

(2) $$y_{ijk}=\mu \;+\;G_{i}\;+\;B_{j(k)}+\;Y_{k}+{\left(GY\right)}_{ik}+\;{\text{ε}}_{ijk}$$

In the two equations (Equations 1 and 2), *y_ijlk_* (or *y_ijk_*) is the phenotype for genotype *i* in block *j*, replication *l*, and year *k*. *μ* is the population mean, *G_i_* is the random effect of genotype *i*, *B_j_* is the random effect of block *j* within an environment, *R_l_* is the random effect of replication *l* within an environment, *Y_k_* is the fixed effect of year *k*, (*GY*)*_ik_* is the genotype *i* by year *k* interaction, and ε*_ijlk_* is the residual error. Each random effect is assumed to be independent from the rest of the random effects and have a normal distribution with mean zero. The newly obtained prediction for the random genotype effects (BLUPs) were then utilized in the ensuing QTL analyses (Park et al., 2021). The mixed model (Equation 1) was exclusively used for length–width and width–depth ratios and tuber weight because the experiment field was composed of two replicated blocks, and 10 tubers from each block were collected. In other words, length–width and width–depth ratios and tuber weight values were collected 10 times from each block. On the other hand, during the data collection processes of tuber shape visual assessment and specific gravity, only one appropriate value was assigned for the 10 selected tubers of each plot. As a result, the second mixed model (Equation 2) was applied to those two traits because the “*R_l_*_(_*_k_*_)_” term in the mixed model (Equation 1) is unnecessary in this situation. A detailed information, labeling system, and distribution patterns of all the obtained BLUP datasets were explored and are discussed in the Result and Discussion sections below.

### Statistical analysis for broad-sense heritability

Broad-sense heritability for each phenotype was calculated using the following equations (Schmidt et al., 2019):


(3) $$H^{2}\;=\;\frac{{\text{σ}}_{g}^{2}}{{\text{σ}}_{p}^{2}}$$

(4) $${\text{σ }}_{p}^{2}\;=\;{\text{σ }}_{g}^{2}+\frac{{\text{σ}}_{gy}^{2}}{y}+\frac{{\text{σ}}_{\varepsilon }^{2}}{y\cdot \beta }$$

In Equation 3, $\sigma_{g}^{2}$ and $\sigma_{p}^{2}$ correspond to the variances in genotypic impact and phenotypic measurements among the replicates, respectively. In Equation 4, $\sigma_{g}^{2}$ , $\sigma_{gy}^{2}$ , and $\sigma_{\varepsilon }^{2}$ represent the variances of the random effect of genotype *i*: *G_i_*, the genotype *i* by year *k* interaction: (*GY*)*_ik_*, and the residual error: ε*_ijk_*, respectively. The terms *y* and *β* used in Equation 4 indicate the numbers of years and blocks, respectively. This study’s statistical analyses and visualization of the resulting data (e.g., histograms) relied on JMP Pro® Statistics, Version 12 (SAS Institute Inc., Cary, NC, USA).

### Correlation tests among the BLUP datasets

The 15 BLUP datasets (**Supplementary Figure 3**), calculated by the two equations (Equations 1 and 2), were then scrutinized by correlation tests to assess consistency across the 2 years within each trait as well as to inspect whether any interrelationship between different traits existed or not. Multivariate function equipped on JMP Pro® Statistics, Version 12 (SAS Institute Inc., Cary, NC, USA) was utilized for all the correlation coefficient test trials. A correlation coefficient was considered statistically significant if its associated correlation probability was below the conventional 5% (p-value< 0.05).

### Genotyping, SNP calling, and dosage evaluation

After extracting DNA samples of the A08241 mapping population, they were genotyped by Illumina Infinium SolCAP SNP array version 3 (21,027 SNPs) and the Illumina iScan system. GenomeStudio software (Illumina, Inc., San Diego, CA, USA) was used to assess DNA quality and to translate the raw genotype data to SNP theta values as described by Park et al. (2019) and Staaf et al. (2008). The theta scores were then converted to typical autotetraploid marker genotypes (AAAA, AAAB, AABB, ABBB, and BBBB) through ClusterCall (version 1.5; R-package) (Schmitz Carley et al., 2017). For access to the marker genotype data, kindly consult Supplementary Material 2 in the publication by Park et al. (2023).

### Linkage group and QTL map construction processes

Thanks to the release of MAPpoly software (v. 0.2.3; R-package), the majority of linkage group assembly became automated. The MAPpoly can examine polyploid organisms up to octoploid when using hidden Markov models (HMM) providing various convenient functions for genetic analyses (Mollinari and Garcia, 2019; Core R. Team, 2020; Mollinari et al., 2020). Once the converted tetraploid SNP markers were loaded on MAPpoly, the *filter_missing*, *filter_segregation*, *make_seq_mappoly*, and *elim.redundant* functions of the software were operated to do primary uninformative marker filtration processes. As explained by Park et al. (2021), the 12 linkage groups were built and refined through two-point analysis, unweighted pair group method with arithmetic mean (UPGMA) hierarchical clustering method, and multidimensional scaling (MDS) embedded in MAPpoly, and the potato reference genome PGSC Version 4.03 (Hackett and Luo, 2003; Potato Genome Sequencing Consortium, 2011; Sharma et al., 2013; Preedy and Hackett, 2016; da Silva Pereira et al., 2020; Mollinari et al., 2020; Spud Database, 2020).

After assembling the 12 linkage groups (refer to Supplementary Figure 2 in Park et al., 2023), the complete linkage maps and phenotype BLUP datasets were loaded on QTLpoly, an R-package developed for automated QTL analysis of polyploid organisms. The *remim* function in QTLpoly first executed a random-effect multiple interval mapping (REMIM) model, fitting various random-effect QTL by evaluating a single parameter per QTL (da Silva Pereira et al., 2020). The QTLpoly then performed linear score statistics tests (Qu et al., 2013) at every position and compared its p-value to a prescribed critical value. The p-values appeared as a continuous pattern over the whole range of the unit interval as a result of weighted sums of the scores from the profiled likelihood (Qu et al., 2013; da Silva Pereira et al., 2020). The continuous p-values were converted to LOP scores by the equation, “LOP = −log10 (p-value)” to delineate and evaluate newly detected QTL in this study intuitively as well as to estimate support intervals of those QTL. The QTL with four or higher LOP scores were decided as significant QTL peaks (da Silva Pereira et al., 2020). Approximately 95% support intervals were used in this study and computed using LOP − 1.5 (Lander and Green, 1987; da Silva Pereira et al., 2020). The *fit_model* argument equipped in QTLpoly was used to calculate the heritability of the significant QTL (da Silva Pereira et al., 2020). The symbol “*h^2^_QTL_*” was used to indicate those QTL heritability values. It should be noted that the QTL heritability (*h^2^_QTL_*) differs from the general heritability (e.g., broad-sense heritability), which represents how well a trait is inheritable from two parents to their progeny. If a significant QTL had over 10% *h^2^_QTL_*, it was considered as a major effect QTL. The reverse case (*h^2^_QTL_* ≤ 10%) was determined to be a minor effect QTL (da Silva Pereira et al., 2020; Park et al., 2021).

### Supplementary accuracy test for the QTL results derived from skewed phenotype data

Since the three BLUP datasets obtained from tuber shape visual assessment phenotype data were skewed (**Supplementary Figure 3**), data transformations using multiple transformation methodologies to normalize distributions were conducted and assessed. The ordered quantile (ORQ) normalization transformation method (Peterson and Cavanaugh, 2020) was determined to be the most effective in normalization of the visual assessment data. After the data transformation, a comparison of the QTL results between transformed and non-transformed data was conducted.

### Analyses of allele effects

The *qtl_effects* function of QTLpoly provided bar graphs revealing allele effects at each identified QTL (**Supplementary Figure 4**). In the allele effect bar graph, the x-axis displayed the four homologs of the two parents. For example, the “a–d” written in **Supplementary Figure 4** stood for four homologs of Palisade Russet, and the “e–h” depicted another four homologs of the ND028673B-2Russ. The y-axis visualized the quantity of an allele effect of each homolog (**Supplementary Figure 4**). This allele effect analysis displayed how much each homolog of the two parents adds to or subtracts from the mean given one of the 190 observed mapping progenies (da Silva Pereira et al., 2020; Park et al., 2021); thus, it was possible to find which allele(s) among the eight homologs of the two parents most significantly impacted a trait. Furthermore, the visually distinguishable high and low bars in those graphs helped efficiently compare the contribution of the two parents to the average of the whole mapping population. Subsequently, the allele effect vectors, which denoted the magnitude of either positive (=increase in) or negative (=decrease in) effect among the four homologs of each parent, were transformed into absolute values. The sum of all the eight absolute values at each mapped locus insinuated the amount of the influence of each mapped significant QTL. Besides, the contribution quantities of each parent for a trait could be assessed based on the sum of each parent’s four absolute values (Park et al., 2021; G. da Silva Pereira, unpublished).

### Conducting single-marker analyses and searching for genes adjacent to the significant QTL provides useful information for the development of diagnostic markers for MAS

In the previous steps, the positional information and characteristics of significant QTL associated with all five traits were investigated. To provide more practically useful information to potato breeders, single-marker analyses (Park et al., 2021) were conducted, and genome sequence coordinates and associated genes adjacent to the SNPs linked to the significant QTL were searched in this study.

The single-marker analysis helps to verify the relationship between differences in SNP alleles and phenotypic changes. This process indirectly assists in confirming whether the allele effects mentioned above (or QTL effects) were actually reflected in the original phenotype data or not and in identifying the most suitable genetic models, such as additive or simplex-dominant models. Once a target QTL and its linked SNP marker were selected, BLUP data were segregated by SNP genotype resulting in two to five distinct genotype groups. Subsequently, the averages of BLUPs for each genotype group were compared to determine significant mean differences between the two genotype groups (Park et al., 2021). The existence of a significant mean difference can indirectly indicate the impact of alleles on the phenotype. For example, if the “B” allele of an SNP marker is linked to an increase in specific gravity and exhibits an additive impact, a higher number of “B” alleles in a genotype would be expected to confer a higher specific gravity. If a genotype group of a certain SNP consists of less than nine individuals (approximately 5% of the total population), it was not considered a comparison group in the single-marker analysis because obtaining statistically reliable results under such conditions is challenging. The one-way analysis of variance (ANOVA) test and Tukey–Kramer mean comparison test (JMP Pro® Statistics, Version 12; SAS Institute Inc., Cary, NC, USA) were employed for this analysis with a significance threshold of p-value< 0.05 (Park et al., 2021).

Finally, all the genome sequence coordinates and associated genes (if available) found within the 200-kilobase (kb) interval surrounding the SNPs linked to the significant QTL were explored (= 100 kb before and after a target SNP). The physical map locations of those SNPs and genome sequence coordinates were obtained from the potato reference genome PGSC v4.03 (Hamilton et al., 2011; Sharma et al., 2013; Uitdewilligen et al., 2013; Spud Database, 2020).

## Results

### Summary of collected phenotype data, their broad-sense heritabilities, and distribution patterns of converted BLUP datasets

#### Length–width ratio and visual assessment of tuber shape

The length–width ratio values of the A08241 mapping population across all tubers collected ranged from 0.99 to 3.49 in 2019 and from 0.98 to 3.82 in 2020. One exception was observed in one of the 20 tubers of A08241-177 in 2020, which had a length–width ratio of 0.77. This outlier ratio was representative of a tuber with a thick middle (width) relative to its length. The mapping population means of the length–width ratio were 1.80 in 2019 and 1.79 in 2020. The average length–width ratios of Palisade Russet across the two blocks were 1.78 in 2019 and 1.64 in 2020, and those of ND028673B-2Russ were 1.91 in 2019 and 1.83 in 2020. Russet Burbank, which was used as a standard control of the russet market class, had 2.16 in 2019 and 2.25 in 2020 as its average length–width ratios.

The visual assessment scores of the two parents in 2019 phenotype data were “5 (long)” persistently across the two blocks. However, in 2020, ND028673B-2Russ was scored as “4 (oblong)” across the two blocks, while Palisade Russet was rated “3 (oval)” and “4” (oblong) in its two replicates. Russet Burbank consistently had the score “5” across the two blocks and across the 2 years. In 2019, the A08241 mapping population did not show the visual assessment score “1 (compressed),” but ranged from “2 (round)” to “5,” but in 2020, all five scales were observed across the mapping population (**Supplementary Figure 2**; **Supplementary Data 1**). When calculating the average visual assessment scores for the A08241 mapping population each year, the obtained values were 4.31 in 2019 and 4.01 in 2020.

The first mixed-effect model (Equation 1) and second mixed-effect model (Equation 2) were used to analyze the length–width ratio and tuber shape visual assessment scores resulting in variance component estimates and BLUP values of the two traits. Variance component estimates of the two traits are summarized in **Table 1**. The comparison of the variance components of the four random effects and the residual of the length–width ratio showed that the clone (or genetic) effect was overwhelmingly more substantial than the other effects occupying 57.91% of the total variance components of the length–width ratio. Likewise, the genetic effect occupied 55.49% of the entire variance components of the visual assessment (**Table 1**). The broad-sense heritabilities of the length–width ratio and visual assessment were 0.83 and 0.81, respectively. It was confirmed that the genetic effect was the primary contributing factor to tuber length and tuber shape in the mapping population.

**Table 1:** Variance component estimates of tuber shape, specific gravity, and tuber weight.

Length–width ratio			Width–depth ratio		
Random effect	Var component^a^	Std error^b^	Random effect	Var component^a^	Std error^b^
Clone^c^	7.76E−02	0.008448	Clone^c^	1.81E−03	0.000214
Block (year]	3.18E−05	5.79E−05	Block (year)	2.83E−05	3.25E−05
Rep (year)	2.03E−04	0.000111	Rep (year)	2.01E−06	7.76E−06
Clone*year	6.73E−03	0.000948	Clone*year	1.05E−04	5.29E−05
Residual	4.91E−02	0.000821	Residual	8.05E−03	0.000134
Total	1.34E−01	0.008484	Total	9.99E−03	0.000251
Fixed effect	Estimate	Std error ^b^	Fixed effect	Estimate	Std error ^b^
Intercept	1.791115	0.021178	Intercept	1.180968	0.004242
Year (2019)	0.005394	0.006501	Year (2019)	−0.00251	0.002917
Visual assessment of tuber shape			Specific gravity		
Random effect	Var component^a^	Std error^b^	Random effect	Var component^a^	Std error^b^
Clone^c^	3.54E−01	0.04566	Clone^c^	3.88E−05	5.61E−06
Block (or rep) (year)	5.02E−03	0.006272	Block (or rep) (year)	1.01E−06	1.18E−06
Clone*year	4.71E−02	0.018894	Clone*year	1.15E−05	3.16E−06
Residual	2.32E−01	0.016988	Residual	3.30E−05	2.42E−06
Total	6.38E−01	0.046798	Total	8.43E−05	5.86E−06
Fixed effect	Estimate	Std error^b^	Fixed effect	Estimate	Std error^b^
Intercept	4.151442	0.059528	Intercept	1.09405	0.000728
Year (2019)	0.151507	0.041089	Year (2019)	0.001636	0.000571
Tuber weight					
Random effect	Var component^a^	Std error^b^			
Clone^c^	1.044186	0.143557			
Block (year)	0.012721	0.014236			
Rep (year)	0.053949	0.020485			
Clone*year	0.4879	0.065381			
Residual	2.842145	0.047492			
Total	4.4409	0.15232			
Fixed effect	Estimate	Std error ^b^			
Intercept	7.396286	0.114016			
Year (2019)	−0.28423	0.086792			

a Variance component.

b Standard error.

c “Clone” indicates a genetic effect of a clone.

After analyzing the raw length–width ratio (LW) and visual assessment (VA) phenotype data with the mixed models (Equations 1 and 2), three different BLUP datasets were produced from each trait, depending on the combination of BLUP effects of each clone. The first BLUP dataset was “LW_clo” and consisted of the BLUPs of pooled phenotypic data across all the 2 years, with the “LW” being an abbreviation for the length–width ratio. The second set, “LW_clo_2019,” was composed of the BLUPs of interaction between a clone and the 2019-year effect. In the same manner, the third set, “LW_clo_2020, contained the BLUPs of interaction between a clone and the 2020-year effect. The same organization and labeling methods were used for the visual assessment phenotype data resulting in the three BLUP datasets, VA_clo, VA_clo_2019, and VA_clo_20202.

Each BLUP dataset introduced above was composed of 184 BLUPs. Even though the 190 progenies were originally prepared and used to develop the 12 linkage maps, several progenies displayed poor field emergence or unhealthy growth during the field test periods, not allowing their inclusion in the analyses. As a result, a total of 184 BLUPs, instead of 190, were included in each BLUP dataset. When distribution patterns of the six tuber shape-related BLUP datasets were visually evaluated, all the LW BLUP datasets (LW_clo, LW_clo_2019, and LW_clo_2020) were close to normal (e.g., a bell shape), but all the three VA BLUP datasets (VA_clo, VA _clo_2019, and VA _clo_2020) revealed some skewness (**Supplementary Figure 3**). Data transformation was conducted with the ORQ normalization transformation method (Peterson and Cavanaugh, 2020) to address the skewed VA data; additional QTL analyses with the transformed data were then conducted to verify whether the observed non-normality of BLUPs influenced the QTL analysis results reported in this study or not. No significant difference was observed in QTL results between transformed and non-transformed data (data not shown) indicating that the level of skewness did not significantly impact the final QTL results. Therefore, this study only considered and analyzed the non-transformed VA BLUP datasets and reported their QTL results.

#### Width–depth ratio

The average width–depth ratios across the two blocks of Palisade Russet were 1.19 in 2019 and 1.20 in 2020, and those of ND028673B-2Russ were 1.17 in 2019 and 1.18 in 2020. Those of the standard control, Russet Burbank, were 1.18 in 2019 and 1.17 in 2020. The width–depth ratio values of the A08241 mapping population were distributed from 0.90 to 1.66 in 2019 and from 0.85 to 1.65 in 2020. The A08241 population mean of the width–depth ratio was 1.18 across the 2 years. Variance component estimates and BLUP datasets of width–depth ratio were obtained through the mixed model (Equation 1). The variance component of the width–depth genetic effect revealed a much bigger value (approximately 18%) compared to those of block[year], rep[year], and clone x year effects, which were close to either 1% or 0% (**Table 1**). Interestingly, unlike the previously shown LW and VA cases, the variance component of residual was the primary contributor (over 80%) to the total observed variance of the width–depth ratio (**Table 1**). The broad-sense heritability of the width–depth ratio was 0.47. Three BLUP datasets of the width–depth ratio were obtained: WD_clo, WD_clo_2019, and WD_clo_20202, with the “WD” being an abbreviation for width–depth ratio. Each dataset was composed of 184 BLUPs as previously described, with WD_clo, WD_clo_2019, and WD_clo_20202 displaying normal distributions (**Supplementary Figure 3**).

#### Specific gravity and tuber weight

The tuber weight of the A08241 mapping population ranged from 25.51 to 601.58 g in 2019 and from 25.80 to 789.25 g in 2020. The population means of the tuber weight were 202.98 g in 2019 and 218.57 g in 2020, respectively. Tuber weight values of Palisade Russet averaged 189.09 g in 2019 and 249.76 g in 2020, respectively. Those of ND028673B-2Russ were 213.19 g in 2019 and 225.95 g in 2020, respectively. The average weight values of Russet Burbank were 218.56 g in 2019 and 250.54 g in 2020. When variance component estimates of tuber weight were compared, genetic (23.51%) and G × E effects (10.99%) were relatively significant compared to block[year] and rep[year] effects (close to either 1% or 0%). As shown in WD case above, the variance component of residual occupied the largest portion (64%) of the total variance of the tuber weight (**Table 1**). The statistical analysis of the tuber weight phenotype data with Equations 3 and 4 resulted in 0.52 as the broad-sense heritability.

The specific gravity values of the A08241 mapping population were distributed from 1.069 to 1.121 in 2019 and from 1.068 to 1.123 in 2020, respectively. The mapping population means of the specific gravity were 1.10 in 2019 and 1.09 in 2020. The average specific gravity values of Palisade Russet and ND028673B-2Russ were 1.104 and 1.088 in 2019 and 1.101 and 1.089 in 2020. The high specific gravity of Palisade Russet has previously been reported (Novy et al., 2012). Those of Russet Burbank were 1.081 in 2019 and 1.083 in 2020. The comparison of the variance component estimates of the specific gravity revealed that the genetic effect (46.03%) was ranked the highest, followed by residual (39.15%) and G × E effect (13.64%). The block[year] effect was close to 1% (**Table 1**). The broad-sense heritability of the specific gravity was 0.73.

Three BLUP datasets were obtained for each specific gravity and tuber weight. The same naming method as introduced above was used with “SG” and “TW” abbreviating specific gravity and tuber weight, respectively, resulting in three BLUP datasets for specific gravity: SG_clo, SG_clo_2019, and SG_clo_2020, and another three BLUP datasets for tuber weight: TW_clo, TW_clo_2019, and TW_clo_2020. The number of components for each BLUP dataset was 184. The distribution patterns of all six BLUP datasets mentioned in this paragraph reflected a normal distribution (**Supplementary Figure 3**).

### Correlation tests of the 15 BLUP datasets within each trait and between different traits

**Figure 1:**
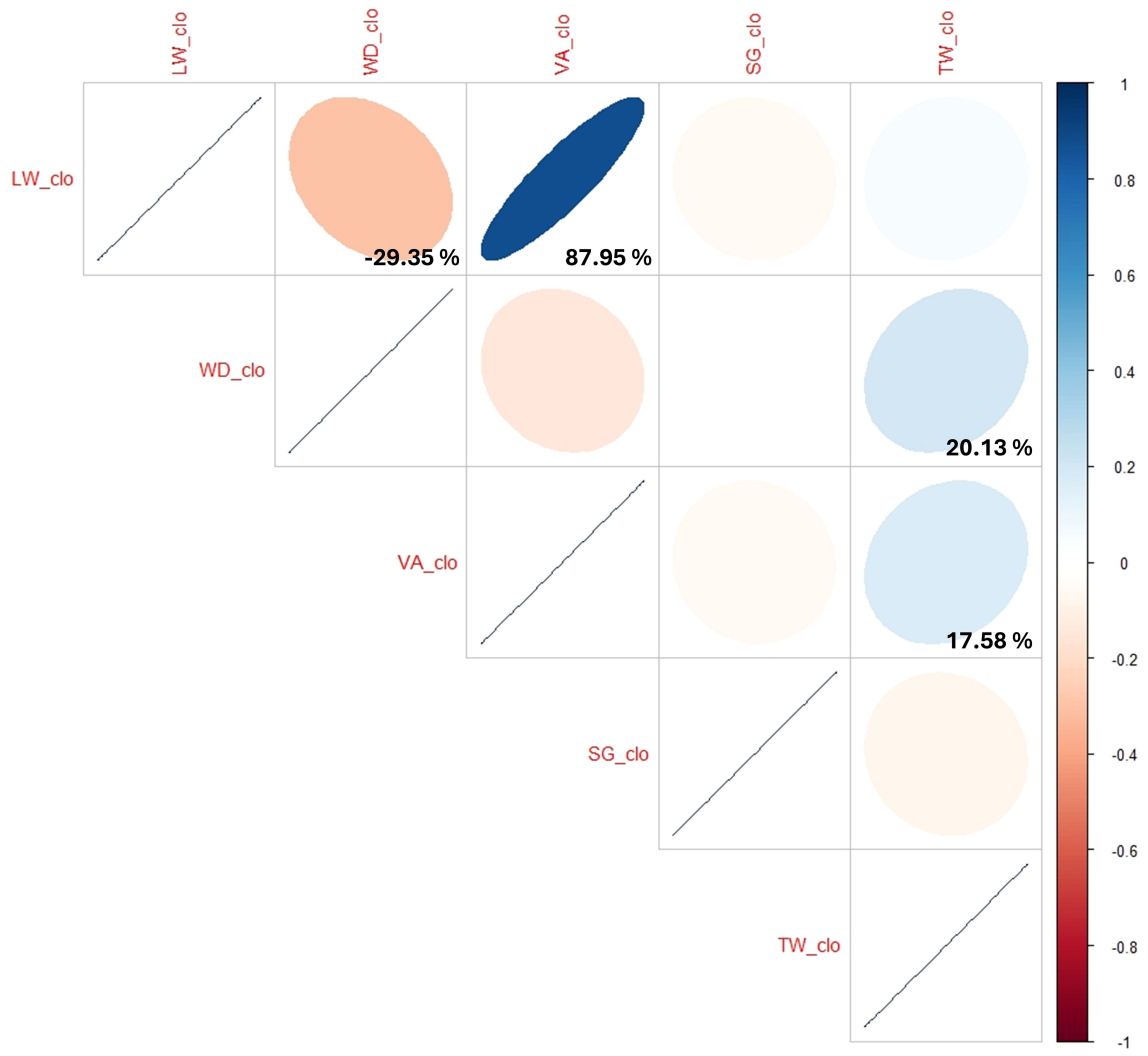
Correlation tests between the pooled phenotypic data of the five traits (LW_clo, WD_clo, VA_clo, SG_clo, and TW_clo).

The correlation tests for the three BLUP datasets within each trait ranged from 73.87% to 99.69% (**Supplementary Table 1**). Overall, no (or minor) variation was observed across the 2-year data regardless of the traits. When the BLUPs of pooled phenotypic data of the five traits (LW_clo, WD_clo, VA_clo, SG_clo, and TW_clo) were compared to each other, the correlation coefficient between LW_clo and VA_clo was the highest (87.95%) followed by the comparison between LW_clo and WD_clo (−29.35%), between WD_clo and TW_clo (20.13%), and between VA_clo and TW_clo (17.58%) (**Figure 1**; **Supplementary Table 1**). Even though the correlation coefficient between WD_clo and VA_clo was −14.17%, it was not statistically reliable due to its higher correlation probability than the threshold (p-value< 0.05). The remainder of those coefficients were basically non-correlated, with values close to 0%.

### Marker selection and linkage mapping processes

The marker selection and linkage mapping processes used in this study were previously developed and detailed by Park et al. (2023), with additional details of the linkage map included in Supplementary Figure 2 of that publication. In brief, 4,040 informative SNP markers were selected for developing the 12 linkage groups. Park et al. (2023) confirmed high accuracy rates and uniform allocation of the selected SNPs across the 12 complete linkage groups with enough supporting evidence.

### QTL for LW, VA, WD, SG, and TW

**Figure 2:**
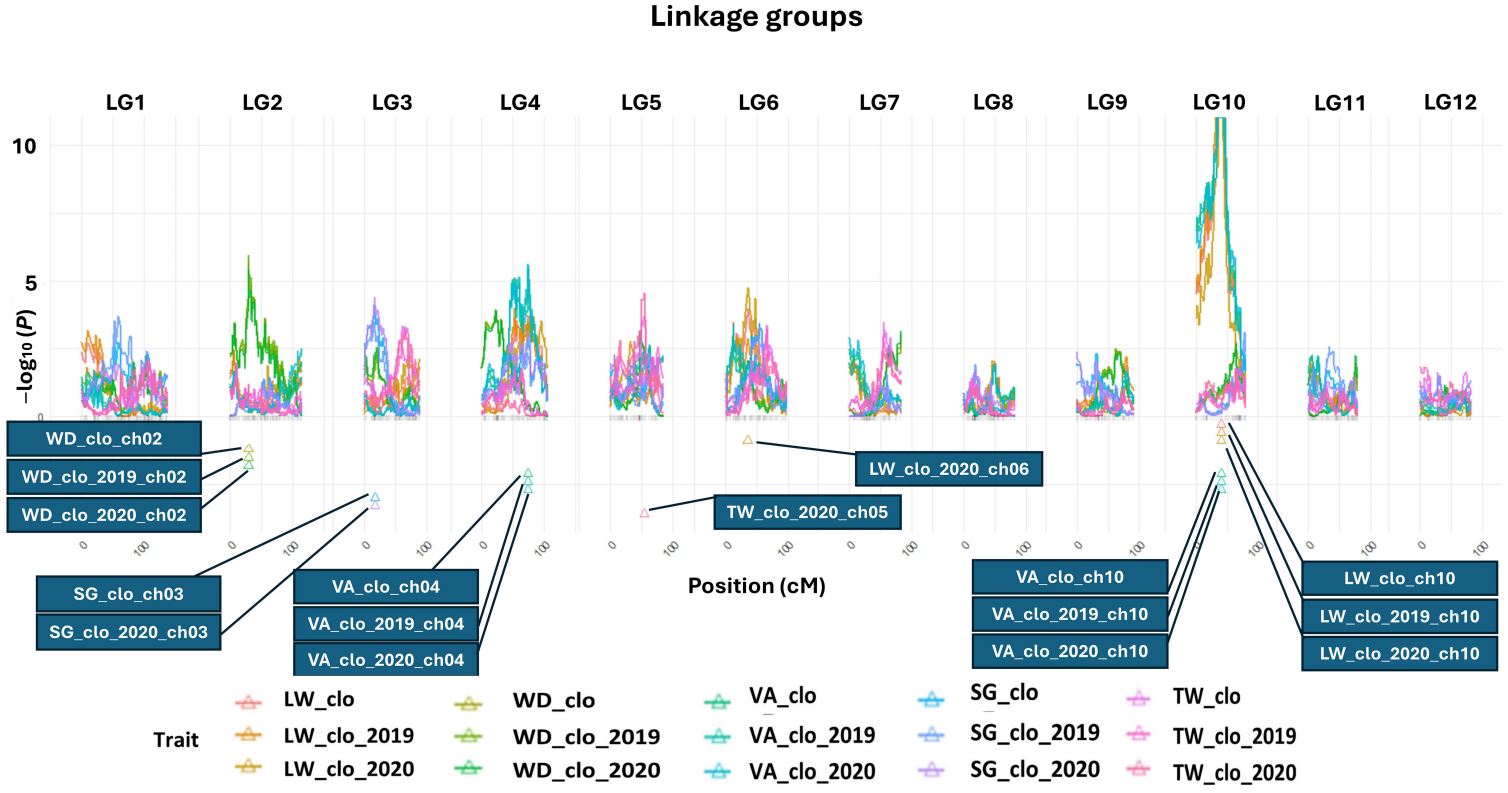
QTL maps for length–width ratio, width–depth ratio, tuber shape visual assessment, specific gravity, and tuber weight. BLUP data abbreviations: LW, length–width ratio; WD, width–depth ratio; VA, tuber shape visual assessment; SG, specific gravity; TW, tuber weight, a genetic effect of clones (clo), 2019 (2019), and 2020 (2020) year effects. Triangles indicate the locations of significant QTL peaks. The x-axis represents 12 different potato chromosomes. The y-axis represents the LOP score, which equals −log10 (p-value). *Panel size limit of the QTLpoly prevented QTL having LOP scores over 11 from being completely visualized on chromosome 10 in this figure. When compared to the peak LOP scores of other QTL (e.g., SG, TW, etc.), the LOP score of the tuber shape QTL peak on chromosome 10 was so extraordinarily high that displaying it alongside the peaks of other QTL became almost impossible.

**Figure 3:**
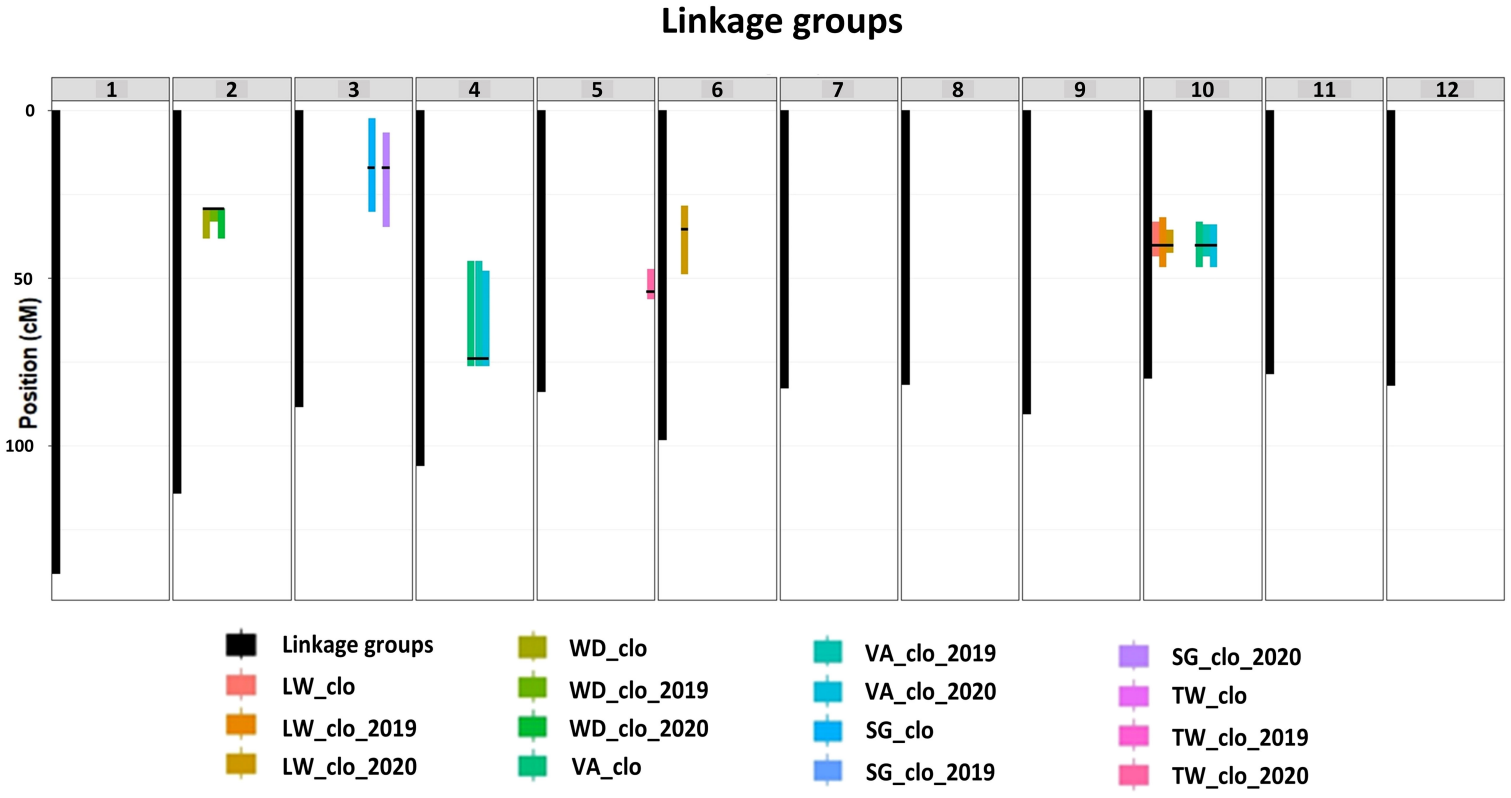
Location information on significant QTL peaks and their support intervals. BLUP data abbreviations: LW, length–width ratio; WD, width–depth ratio; VA, tuber shape visual assessment; SG, specific gravity; TW, tuber weight, a genetic effect of clones (clo), 2019 (2019), and 2020 (2020) year effects. The x-axis represents 12 different potato chromosomes. Black bars indicate the length of each chromosome. Color bars indicate the length of each support interval. Black thin horizontal lines on each support interval indicate the locations of the mapped significant QTL peaks.

All the QTL analysis results discussed below are organized in **Figures 2**, **3** and **Table 2** presenting LOP scores, locations [including both chromosome number and exact position in centiMorgan (cM)], support intervals, QTL heritability (*h^2^_QTL_*), and proximate SNP markers to the mapped QTL.

**Table 2:** Summary table of QTL for tuber shape, specific gravity, and tuber weight.

QTL titles	BLUP datasets^a^	Chr^b^	LOP Score	Heritability of mapped QTL (*h^2^_QTL_*)	QTL position (support interval) (Unit: cM)^c^	Closest marker (physical map position)^d^
*LW_clo_ch10*	*LW_clo*	10	>15.65^e^	0.51	**40.05** (33.18–43.44)	**c2_25471^1)^** (48808404)
*LW_clo_2019_ch10*	*LW_clo_2019*	10	>15.65^e^	0.54	**40.05** (32.04–46.49)	**c2_25471^1)^** (“)
*LW_clo_2020_ch10*	*LW_clo_2020*	10	>15.65^e^	0.42	**40.05** (35.64–42.32)	**c2_25471^1)^** (“)
*LW_clo_2020_ch06*	*LW_clo_2020*	6	4.76	0.14	**35.44** (28.57–48.62)	**c2_31648** (40171177)
*VA_clo_ch10*	*VA_clo*	10	>15.65^e^	0.47	**40.05** (33.18–46.49)	**c2_25471 ^1)^** (48808404)
*VA_clo_2019_ch10*	*VA_clo_2019*	10	>15.65^e^	0.45	**40.05** (34.16–43.44)	**c2_25471 ^1)^** (“)
*VA_clo_2020_ch10*	*VA_clo_2020*	10	>15.65^e^	0.47	**40.05** (34.16–46.49)	**c2_25471 ^1)^** (“)
*VA_clo_ch04*	*VA_clo*	4	5.49	0.12	**74.04** (45.06–76.13)	**PotVar0075244** (67806918)
*VA_clo_2019_ch04*	*VA_clo_2019*	4	5.07	0.11	**74.04** (45.06–76.13)	**PotVar0075244** (“)
*VA_clo_2020_ch04*	*VA_clo_2020*	4	5.60	0.13	**74.04** (47.83–76.13)	**PotVar0075244** (“)
*WD_clo_ch02*	*WD_clo*	2	5.72	0.35	**29.20** (29.20–38.16)	**c2_41980** (27557527)
*WD_clo_2019_ch02*	*WD_clo_2019*	2	5.95	0.35	**29.20** (29.20–32.88)	**c2_41980** (“)
*WD_clo_2020_ch02*	*WD_clo_2020*	2	5.42	0.34	**29.20** (29.20–38.16)	**c2_41980** (“)
*SG_clo_ch03*	*SG_clo*	3	4.13	0.18	**17.05** (2.38–30.12)	**c1_3348 ^2)^** (16008572)
*SG_clo_2020_ch03*	*SG_clo_2020*	3	4.40	0.18	**17.05** (6.67–34.61)	**c1_3348 ^2)^** (“)
*TW_clo_2020_ch05*	*TW_clo_2020*	5	4.56	0.22	**54.06** (47.34–56.03)	**c2_50176** (43712364)

a The names of the BLUP datasets used for each QTL analysis.

b Chromosome numbers.

c The bold figures indicate the locations of the mapped QTL peaks and numbers in the parentheses showing ranges of their support intervals. The unit is centiMorgans (cM).

d The most adjacent SNPs to each QTL peak were presented in this column; “solcap_snp_” was omitted at the beginning of all the SNP marker names beginning with either “c1” or “c2.”

The numbers inside the parentheses represent physical map locations of each SNP (PGSC v4.03).

e The maximum LOP score, which can be reported by QTLpoly software, is 15.65. As a result, “>15.65” represents a higher number than the 15.65 LOP scores in this table.

^1)–2)^If more than one SNP marker are located at the same position, the rest of the SNPs are written below.

^1)^solcap_snp_c2_25471 (48808404), solcap_snp_c2_25469 (48808653), solcap_snp_c1_8021 (48862950), solcap_snp_c1_8020 (48863048), and solcap_snp_c1_8019 (48863165).

The numbers inside the parentheses represent physical map locations of each SNP (PGSC v4.03).

^2)^solcap_snp_c1_3348 (16008572), solcap_snp_c1_10725 (16833849), solcap_snp_c1_10734 (16843656), PotVar0121932 (16887160), and PotVar0121927 (16887361).

The numbers inside the parentheses represent physical map locations of each SNP (PGSC v4.03).

#### QTL for LW

The most significant QTL for the LW consistently emerged at 40.05 cM on chromosome 10 across the three LW BLUP datasets (**Figure 2**; **Table 2**). The three support intervals of the LW_clo_ch10, LW_clo_2019_ch10, and LW_clo_2020_ch10 QTL commonly shared the zone between 35.64 and 42.32 cM (**Figure 3**; **Table 2**). All of their LOP scores were higher than the software maximum limit, 15.65, and their QTL heritabilities (*h^2^_QTL_*) were also high ranging from 0.42 to 0.54. The closest SNP marker to the three QTL was solcap_snp_c2_25471 (**Table 2**).

Additionally, another QTL was detected at 35.44 cM on chromosome 6 with the LW_clo_2020 BLUP data. This QTL did not appear from the other two QTL analyses for LW_clo and LW_clo_2019 BLUP datasets. Its LOP score was 4.76 with 0.14 *h^2^_QTL_*. The support interval of the QTL occupied from 28.57 to 48.62 cM. The most adjacent marker was solcap_snp_c2_31648 (**Table 2**).

#### QTL for VA

The QTL analysis results of VA are similar to those of the LW QTL analyses. For instance, the significant QTL at 40.05 cM on chromosome 10 identified with the LW analyses was also identified with the VA BLUP datasets. The most proximal SNP marker is solcap_snp_c2_25471 again. Likewise, the support intervals of the three QTL (VA_clo_ch10, VA_clo_2019_ch10, and VA_clo_2020_ch10) commonly occupied from 34.16 to 43.44 cM, and their LOP scores were higher than 15.65. Their *h^2^_QTL_* also showed similar values (~0.47) to those observed with the LW BLUP datasets (**Table 2**).

Additionally, another significant QTL was observed on chromosome 4 at 74.04 cM across the three VA BLUP datasets (**Figure 2**; **Table 2**). The support intervals of the three QTL commonly shared the area between 47.83 and 76.13 cM (**Figure 3**; **Table 2**). Their LOP scores and *h^2^_QTL_* were averagely 5.39 and 0.12, respectively. The closest marker to the QTL was PotVar0075244.

#### QTL for WD

QTL analysis for WD ratio resulted in the identification of a significant QTL on chromosome 2 based on the three WD BLUP datasets (**Figures 2**, **3**; **Table 2**). The three QTL (WD_clo_ch02, WD_clo_2019_ch02, and WD_clo_2020_ch02) were located at 29.20 cM on chromosome 2, and their support intervals ranged from 29.20 to 38.16 cM (**Figure 3**; **Table 2**). The average LOP scores and *h^2^_QTL_* of the three QTL were approximately 5.70 and 0.35, respectively. The most adjacent SNP to the QTL position was solcap_snp_c2_41980.

#### QTL for SG and TW

Two significant and apparently identical QTL for SG were detected in the SG_clo and SG_clo_2020 BLUP datasets. The QTL was located on chromosome 3 at 17.05 cM. The support intervals were between 2.38 and 34.61 cM across the two BLUP datasets with LOP scores of 4.13 and 4.40 for SG_clo_ch03 and SG_clo_2020_ch03 QTL, respectively; *h^2^_QTL_* was 0.18. The closest marker to the QTL was solcap_snp_c1_3348. No significant QTL was found from the use of the SG_clo_2019 BLUP dataset (**Figures 2**, **3**; **Table 2**).

One significant QTL for TW was detected on chromosome 5 at 54.06 cM (**Figure 2**; **Table 2**). Its closest SNP was solcap_snp_c2_50176. The LOP score and *h^2^_QTL_* of the QTL were 4.56 and 0.22, respectively.

#### Allele effects of the mapped QTL

The results of all the allele effect analyses are presented in **Supplementary Figure 4** and **Supplementary Table 2**. Interestingly, Palisade Russet consistently showed significantly higher contributions in tuber shape traits (both LW and VA) than ND028673B-2Russ. Furthermore, unlike the other four traits, only TW was more affected by ND028673B-2Russ than Palisade Russet. Other details, such as the most impactful homolog for each trait, will be discussed in the following Discussion section.

##### Exploring SNPs linked to the significant QTL through single-marker analysis and reference genome.

Following the exploration of all SNPs listed in **Table 2** using the potato reference genome PGSC v4.03, the genomic coordinates and associated genes located within the 200-kb interval around these SNPs are arranged in **Supplementary Table 3** providing useful information for MAS.

Conducting single-marker analysis allowed for the evaluation of changes in the tested traits based on the presence (or absence) of an allele of SNPs linked to a QTL. Significant mean differences between genotype groups were identified for the following SNP markers: solcap_snp_c2_25471, solcap_snp_c2_25469, solcap_snp_c1_8021, solcap_snp_c1_8020, and solcap_snp_c1_8019 in relation to both LW and VA. Additionally, solcap_snp_c2_31648 showed significance only for LW, PotVar0075244 only for VA, solcap_snp_c2_41980 for WD, and finally, both solcap_snp_c1_10725 and PotVar0121927 for SG (**Supplementary Figure 5**).

## Discussion

### Exploring the correlation coefficients between years within each trait and across different traits

The correlative analyses for the three BLUP datasets within each trait consistently showed high or extremely high correlation coefficients indicating little variability between the 2 years of phenotypic data collected for each trait (**Supplementary Table 1**). A relatively lower correlation (73.87%) was observed between TW_clo_2019 and TW_clo_2020 as an exception. According to Gebreselassie et al. (2016), tuber weight (=tuber size in this case) can be significantly impacted by environmental effects or G × E.

Since all the correlations across the 2 years within each trait were generally quite high, the BLUPs of pooled phenotypic data of the five traits (e.g., LW_clo, WD_clo, VA_clo, SG_clo, and TW_clo) were picked and then compared to each other through correlation tests. As expected, the correlation coefficient between LW_clo and VA_clo revealed the highest value (87.95%) compared to others because the two different methods assessed tuber length as a main factor. Interestingly, WD_clo showed a minor correlation with LW_clo (−29.35%) and TW_clo (20.13%) (**Figure 1**; **Supplementary Table 1**). This suggests that the degree of tuber flatness is not influenced to a great degree by tuber length or size.

The relationship between tuber shape and tuber size (=TW_clo) could not be clarified in the correlation test. This was because the correlation coefficient between LW_clo and TW_clo was close to 0%, and its probability was much higher than the threshold (p-value< 0.05). On the other hand, a correlation coefficient of 17.58% was observed between VA_clo and TW_clo. This contradiction could be partly explained by the skewness observed in VA_clo. Unlike the LW_clo, VA_clo was significantly skewed toward a positive effect (**Supplementary Figure 3**). It was assumed that during the visual assessment process, many large tubers belonging to category 5, even if their size and LW were the same or similar to other tubers belonging to category 4, had the effect of gaining one point more resulting in the skewness in all the three VA BLUP datasets (**Supplementary Figures 2** and **3**). Therefore, caution is warranted in interpreting that tuber size (e.g., TW_clo) and VA_clo were related by solely relying on the 17.58% correlation coefficient. Therefore, this study tentatively concluded that the A08241 population did not show a meaningful relationship between tuber shape and tuber size based on the poor correlation coefficient of the LW_clo and TW_clo BLUP datasets, which were numerical and much less likely impacted by human bias during assessment.

### The most predominant tuber shape QTL on chromosome 10

Chromosome 10 was identified as having a major QTL that impacted tuber shape. Regardless of tuber shape measurement methods (digital caliper vs. naked eyes), a major QTL for both LW and VA consistently appeared at 40.04 cM, having the maximum software LOP scores, and high QTL heritability (*h^2^_QTL_*) reaching approximately 50% (**Table 2**). In the allele effect analysis for this position (40.05 cM on chromosome 10), Palisade Russet contributed at least 65% or more, compared to ND028673B-2Russ, across all the LW and VA QTL (**Supplementary Table 2**). Interestingly, Palisade Russet had both the most positive (=causing longer shape) and negative (=causing rounder shape) effects at homologs c and d, respectively. The most negative allele effect tended to be consistently stronger than the most positive effect across the LW_clo_ch10, LW_clo_2019_ch10, and LW_clo_2020_ch10 QTL (**Supplementary Figure 4**; **Supplementary Table 2**). During the single-marker analysis, all five SNPs (**Table 2**) linked to both LW and VA QTL consistently showed statistically significant differences in the progeny’s LW and VA BLUP dataset means according to genotype groups implying an additive genetic model as well as providing practical information for future MAS. For example, while analyzing solcap_snp_c2_25471, there was a statistically significant tendency for a progeny to have a greater negative impact (round shape) as it possessed more “B” alleles (**Supplementary Figures 5a_1, b_1**). In this way, using the five SNPs (solcap_snp_c2_25471, solcap_snp_c2_25469, solcap_snp_c1_8021, solcap_snp_c1_8020, and solcap_snp_c1_8019) for future MAS for tuber shape seems to be a quite promising strategy (**Supplementary Figures 5a_1–a_5, b_1– b_5**).

The identification of a major QTL on chromosome 10 influencing tuber shape confirms the findings of Park et al. (2021), who similarly identified a major QTL on chromosome 10 using a different tetraploid mapping population derived from Rio Grande Russet and Premier Russet. By comparing the location information of the two linked SNP markers and various references reporting genes or QTL affecting tuber appearances, Park et al. (2021) identified the Ro locus, known to confer a round shape dominant-to-longer form, as the candidate gene for the tuber shape QTL on chromosome 10 (Masson, 1985; Sharma et al., 2013; Hirsch et al., 2014; Endelman and Jansky, 2016; Hara-Skrzypiec et al., 2018; Spud Database, 2020). The two linked SNPs reported by Park et al. (2021) were compared with the five SNPs (**Table 2**) linked to the tuber shape QTL identified in this study using the potato reference genome PGSC Version 4.03 (Sharma et al., 2013; Spud Database, 2020). As expected, those linked SNPs between the two studies were closely placed showing 1 cM as an average distance between them. Almost identical results were also observed from the additional comparison tests with the other tuber appearance-related QTL identified by previous studies relying on diploid mapping populations experimented in different environments (Endelman and Jansky, 2016; Hara-Skrzypiec et al., 2018). Overall, the Ro locus, located approximately 40.05 cM on chromosome 10 (**Table 2**), appears to have the most substantial impact on tuber length in potato regardless of genetic backgrounds, ploidy levels, and environmental conditions. This finding can be utilized in developing diagnostic molecular markers useful to potato breeders in selecting long (russet market class) or round (chipper market class) tuber type.

### QTL analysis for VA gave rise to unpredictable QTL on chromosome 4

One of the purposes of this study was to assess whether the distinct features between LW and VA measurement methods of tuber shape significantly affected the final QTL analysis results or not. As discussed above, one primary tuber shape locus at 40.05 cM on chromosome 10 harbored all the six QTL (e.g., LW_clo_ch10, LW_clo_2019_ch10, LW_clo_2020_ch10, VA_clo_ch10, VA_clo_2019_ch10, and VA_clo_2020_ch10) with very similar LOP scores and QTL heritabilities (*h^2^_QTL_*) with each other. Those QTL results proved that the difference in tuber shape evaluation systems did not significantly affect the localization process of the Ro locus and the appraisal of its major effect on tuber shape.

Of interest was the identification of additional QTL-impacting tuber shape at 74.04 cM on chromosome 4 with the VA_clo, VA_clo_2019, and VA_clo_2020 BLUP datasets. It is important to note that no QTL was detected at the same position on chromosome 4 during all the QTL analyses with all three LW BLUP datasets. Compared to the tuber shape QTL on chromosome 10, the QTL found on chromosome 4 had relatively lower LOP scores, but their LOP scores were still higher enough to be considered significant with influential *h^2^_QTL_* (up to 13%) (**Table 2**). To validate the impact of the skewness in the original VA BLUP datasets on VA_clo_ch04, VA_clo_2019_ch04, and VA_clo_2020_ch04, the QTL outcomes of the non-transformed VA BLUP datasets were juxtaposed with those of the ORQ normalization-transformed VA BLUP datasets (Peterson and Cavanaugh, 2020). No statistically meaningful distinction was detected (data not shown) like the three VA QTL on chromosome 10 mentioned above. Thus, only non-transformed VA BLUP datasets and their QTL results were considered here. The allele effect analysis for this position (74.04 cM on chromosome 4) disclosed that Palisade Russet contributed higher impacts ranging from 52% to 63% than ND028673B-2Russ across the three VA BLUP datasets (**Supplementary Table 2**). The most positive (=causing longer shape) and negative (=causing rounder shape) effects were located on homologs d and c of Palisade Russet, respectively (**Supplementary Figure 4**; **Supplementary Table 2**).

During the single-marker analysis for PotVar0075244, the genotype groups with more “B” alleles tended to exhibit a stronger positive effect resulting in longer tubers (**Supplementary Figure 5b_6**). However, a statistically significant difference was observed only in the comparison between the AAAB and ABBB genotype groups (**Supplementary Figure 5b_6**). Unlike the five SNPs linked to the major tuber shape QTL on chromosome 10, relying solely on the PotVar0075244 marker to achieve MAS for tuber shape does not seem promising.

According to the potato reference genome PGSC Version 4.03, the SNP marker, PotVar0075244, linked to QTL VA_clo_ch04, VA_clo_2019_ch04, and VA_clo_2020_ch04, was at the end of the PGSC0003DMG400008004 genome sequence coordinate, which putatively represented the granulin repeat cysteine protease family of proteins (Hamilton et al., 2011; Sharma et al., 2013; Uitdewilligen et al., 2013; Spud Database, 2020). In multiple prior research projects that evaluated SPCP3 protein and its homologies observed in potato, sweet potato, tomato, soybean, and *Arabidopsis*, it was discovered that the protein is associated with physiological changes in plants, including programmed cell death in leaves and hypersensitive reactions triggered by pathogens. Additionally, SPCP3 is suggested to be a precursor protein for a plant granulin-containing cysteine protease implying the potential involvement of cysteine proteases in the potato tuber formation, which is also one of the potato physiological phenomena (Avrova et al., 1999; Kamoun et al., 1999; Chen et al., 2006). Weeda et al. (2009) monitored changes in cysteine protease activities and a multidomain cysteine protease inhibitor during potato plant development as well as tuber formation. Interestingly, they observed that the cysteine protease’s activity rate and the associated inhibitor’s concentration started to change significantly in stolons from the beginning of tuber formation and confirmed that those metabolic processes were linked to regulating tuber protein content *in vivo* (Weeda et al., 2009). In this current study, it could be hypothesized that potato tuber shape could be affected by the activity of one of the cysteine protease family of genes based on the close proximity of the three VA QTL on chromosome 4 to PGSC0003DMG400008004 and the findings of Weeda et al. (2009) that cysteine protease activity appeared to be associated with tuberization.

The finding of a major QTL on chromosome 4 using the three VA BLUP datasets, with no QTL detected at that same region using the LW BLUP datasets, was unexpected. This finding may relate to the capability of VA to read and reflect tuber shoulder shape (the regions near the ends of the tuber that can flare out before ending in a point). If two population clones have divergent shoulder silhouettes but have the same LW, they might not be clearly distinguished during the LW measurement even though the VA assessment can sort them into two different categories. For example, let us assume that there are two different tubers: oblong (relatively cylindrical shape: VA score 4 of **Supplementary Figure 2**) and long (relatively pointy at each end: VA score 5 of **Supplementary Figure 2**) tubers having the same LW. The two tubers would not be distinguishable in LW but be discernible in VA. We suspect that the ability to catch shoulder silhouettes in the tuber may have contributed to the identification of a QTL on chromosome 4 using the VA assay, which was not identified using the LW protocol. Another possible scenario is that contrary to the simple two-dimensional image data obtained by LW measurement, human eyes generally accept visual information as a three-dimensional image, covering the length of the tubers as well as other factors such as regularity among the tested tubers of each clone, which may affect an evaluator’s decisions in assigning a shape category using VA. This may then have contributed to the identification of the significant QTL on chromosome 4, which was not identified in the LW analyses of tuber shape.

Concurrently, even though this current study and Park et al. (2021) found significant QTL for tuber shape on chromosome 4, those QTL were approximately 48 cM away from each other, confirming that each QTL from the two studies likely represented different genes (Hamilton et al., 2011; Sharma et al., 2013; Uitdewilligen et al., 2013; Spud Database, 2020). It would seem, however, that chromosome 4 appears to contribute to tuber shape based on this study and previous QTL analyses for tuber shape.

### A significant QTL for LW exclusively obtained from LW_clo_2020 BLUP

Interestingly, a QTL analysis with the LW_clo_2020 BLUP dataset also resulted in a significant QTL on chromosome 6, which was not observed with the VA assessment. The single-marker analysis for solcap_snp_c2_31648 linked to LW_clo_2020_ch06 QTL showed significant mean difference between two observed genotype groups demonstrating its sufficient influence reflected in the phenotype data (**Supplementary Figure 5a_6**). When tracing the location information on solcap_snp_c2_31648 (**Table 2**), based on the potato reference genome PGSC Version 4.03, it was located in the middle of the PGSC0003DMG400016314 genome sequence coordinate. This coordinate had DNA sequences for an unknown conserved gene, whose function has not yet been studied (Hamilton et al., 2011; Sharma et al., 2013; Uitdewilligen et al., 2013; Spud Database, 2020). Although it was difficult to identify the candidate genes of the LW_clo_2020_ch06 QTL, it could be inferred that an unknown gene influencing tuber shape may exist near 35.44 cM on chromosome 6 (**Table 2**), and this gene seems to be significantly affected by environmental conditions, as it only appeared in the data from the year 2020. When checking the 200-kb interval around the solcap_snp_c2_31648, 14 genome sequence coordinates were additionally observed (**Supplementary Table 3**). Studying the genes associated with the 14 genome sequence coordinates together might help elucidate the nature of the LW_clo_2020_ch06 QTL.

### Significant QTL for tuber depth were located on chromosome 2

Unlike most previous genetic studies covering potato tuber shape (Śliwka et al., 2008; Bradshaw et al., 2008; Prashar et al., 2014; Endelman and Jansky, 2016; Hara-Skrzypiec et al., 2018; Manrique-Carpintero et al., 2018; Meijer et al., 2018; Park et al., 2021), this study was unique in also assessing tuber depth (flatness of the tuber). Since the evaluated tubers’ sizes ranged from 25.51 to 789.25 g, the width–depth (WD) ratio provided a more intuitive assessment than depth information alone. For instance, if a WD value is much higher than others, then a flat tuber analogous to a hamburger patty can be readily imagined and vice versa, regardless of tuber length.

Chromosome 2 consistently displayed significant QTL at 29.20 cM using all three datasets (WD_clo_ch02, WD_clo_2019_ch02, and WD_clo_2020_ch02) (**Figure 2**; **Table 2**). The LOP scores (~5.70) and *h^2^_QTL_* (~35%) of the QTL make it an important region for its impact on tuber depth. In the allele effect analysis for this position (29.20 cM on chromosome 2), it was revealed that Palisade Russet and ND028673B-2Russ contributed 51.5% to 53.5% and 46.5% to 48.5%, respectively, across the three WD BLUP datasets. Among the eight allele effects, the most powerful positive effect, which tended to flatten the tuber, was detected on homolog d of Palisade Russet (**Supplementary Figure 4**; **Supplementary Table 2**). In the single-marker analysis for solcap_snp_c2_41980 linked to all the three WD QTL, even though the mean of each genotype group decreased in an additive fashion as the number of “B” alleles in each genotype group increased, consistent significant difference across the three WD BLUP datasets was only observed from the comparison between AABB and BBBB genotype groups (**Supplementary Figure 5c**). To achieve a more robust MAS for tuber depth, it seems necessary to make efforts in searching for additional molecular markers, along with solcap_snp_c2_41980, to create a more reliable diagnostic marker set, such as a haplotype genetic marker. The solcap_snp_c2_41980 SNP marker was located in the center of PGSC0003DMG400010438 genome sequence coordinate. Referring to the genome sequence coordinate information and other references, it was revealed that the coordinate included the sequence of the sulfiredoxin (Srx) gene, which has been known to be involved in oxidation stress resistance in yeast, human, and *Arabidopsis* (Biteau et al., 2003; Chang et al., 2004; Basu and Koonin, 2005; Liu et al., 2006; Hamilton et al., 2011; Sharma et al., 2013; Uitdewilligen et al., 2013; Spud Database, 2020). According to our best knowledge, any connection between the Srx gene and potato tuber appearance (or potato cell cycle) has not been researched yet; thus, it is not easy to conclude whether the Srx gene affects tuber depth or another unknown gene(s) involved in tuber depth exists in (or near) PGSC0003DMG400010438. Within the 200-kb interval surrounding solcap_snp_c2_41980, an additional 20 genome sequence coordinates have been found (**Supplementary Table 3**). Further research using advanced fine mapping or direct tests to evaluate the contribution of the Srx gene (or other adjacent genes) to tuber appearance is necessary to resolve this question.

### Significant QTL for SG were detected on chromosome 3

QTL analyses with SG_clo and SG_clo_2020 BLUP datasets gave rise to SG_clo_ch03 and SG_clo_2020_ch03 QTL, respectively, at 17.05 cM on chromosome 3. Their LOP scores and QTL heritabilities (*h^2^_QTL_*) were high enough to be considered as significant QTL (**Table 2**). Even though the QTL analysis with the SG_clo_2019 BLUP dataset did not produce significant QTL, the overall shape of its QTL map on chromosome 3 was similar to those of the SG_clo and SG_clo_2020 BLUP datasets (**Figure 2**). In the allele effect analysis for this position (17.05 cM on chromosome 3), Palisade Russet contributed 50.4% of SG_clo_ch03 QTL and 55.5% of SG_clo_2020_ch03 (**Supplementary Table 2**). The two major negative-effect alleles (=lowering SG) were located at homologs b and g revealing a more substantial impact, approximately twice (or more) than other positive allele effects (**Supplementary Figure 4**; **Supplementary Table 2**). In single-marker analyses targeting the five SNPs (solcap_snp_c1_3348, solcap_snp_c1_10725, solcap_snp_c1_10734, PotVar0121932, and PotVar0121927) linked to SG_clo_ch03 and SG_clo_2020_ch03 QTL (**Table 2**), only solcap_snp_c1_10725 and PotVar0121927 consistently showed a significant mean difference across the two BLUP datasets (SG_clo & SG_clo_2020) in one of their genotype group comparisons (**Supplementary Figures 5d_1, d_2**). To practically utilize the two SNP markers in future potato breeding programs, further research appears to be necessary. The solcap_snp_c1_10725 and solcap_snp_c1_10734 were located on (or near) the PGSC0003DMG400016922 genome sequence coordinate associated with the glycine-rich RNA-binding protein (Hamilton et al., 2011; Sharma et al., 2013; Uitdewilligen et al., 2013; Spud Database, 2020). Li et al. (2019) studied the effect of external glycine on starch biosynthesis in sweet potato (*Ipomoea batatas* Lam.) confirming that applying a low glycine stimulus enhances starch biosynthesis in storage roots by accelerating carbohydrate metabolism and regulating the expression of genes related to starch. Therefore, it appears necessary to verify whether the glycine-rich RNA-binding protein actually has a significant impact on potato starch formation and tuber specific gravity. PotVar0121932 and PotVar0121927 were located on PGSC0003DMG400016921, which is related to histone H2B. Although Mali et al. (2023) conducted gene expression profiling of the potato *JMJ* gene family histone demethylases (*StJMJs*) in stolon tissues of heat-sensitive and heat-tolerant genotypes under elevated temperature revealing that *StJMJs* play a crucial role as epigenetic regulators influencing heat tolerance in potatoes, to the best of my knowledge, there is no clear research on the correlation between histone H2B and tuber dry matter formation. The solcap_snp_c1_3348 SNP marker was observed near the PGSC0003DMG400013960 genome sequence coordinate on chromosome 3. This locus seemed to have the DNA sequence of a gene, but the gene’s function has not been investigated yet (Hamilton et al., 2011; Sharma et al., 2013; Uitdewilligen et al., 2013; Spud Database, 2020). In addition to the previously mentioned three genome sequence coordinates, 12 additional genome coordinates were discovered within the 200-kb intervals of the five SNPs (**Supplementary Table 3**). Exploring the zone where SG_clo_ch03 and SG_clo_2020_ch03 QTL were placed will be helpful in both developing a diagnostic molecular marker linked to SG and identifying which gene(s) significantly influence specific gravity.

Park et al. (2021) identified multiple QTL associated with specific gravity on chromosomes 1 and 5, but none were identified for chromosome 3. Park et al. (2021) utilized a mapping population also derived from two russet-type tetraploid parents (Rio Grande Russet and Premier Russet), with the field analyses in Idaho also conducted at the USDA-ARS Small Grains and Potato Germplasm Research Unit (Aberdeen, ID), but in 2010 and 2011. The differing genetics of the two russet mapping populations and the environmental impacts of divergent years likely contributed to the disparate QTL observed between this study and that of Park et al. (2021). However, other studies have identified and reported QTL or loci associated with SG or tuber starch content (which can be interpreted as a characteristic of specific gravity) on chromosome 3 (Freyre and Douches, 1994; Schäfer-Pregl et al., 1998; Li et al., 2008; Schönhals et al., 2016). Therefore, based on previous studies and this current study, it appears that environmental and G × E effects, as well as the contribution of multiple loci likely impact specific gravity with no clear major loci having yet been identified. Stevenson et al. (1954), Johansen et al. (1967), and Ruttencutter et al. (1979) commonly observed variations in specific gravities (or dry matters) occurring in particular cultivars or breeding clones as environmental conditions (e.g., locations, years, irrigation, etc.) change additionally supporting the involvement of environment and G × E effects in SG.

### Significant QTL for TW was detected on chromosome 5

Among the three TW BLUP datasets, only TW_clo_2020 BLUPs produced a significant QTL (TW_clo_2020_ch05) at 54.06 cM on chromosome 5. The LOP score and QTL heritability (*h^2^_QTL_*) were 4.56% and 22%, respectively. Interestingly, Bradshaw et al. (2008) also identified a QTL linked to tuber size using a mapping population obtained from the cross between 12601ab1 and Stirling. However, the physical map location information for the QTL was unavailable preventing a direct comparison with the TW_clo_2020_ch05 QTL discovered in this study. The allele effect analysis for the position (54.06 cM on chromosome 5) revealed that ND028673B-2Russ contributed 65.0% of TW_clo_2020_ch05 QTL (**Supplementary Table 2**). The most positive allele effect (=increasing tuber weight) was located at homolog g and was approximately 18% bigger than the most negative allele effect (=lowering tuber weight) placed on homolog f (**Supplementary Figure 4**; **Supplementary Table 2**). The closest SNP to the TW_clo_2020_ch05 QTL was solcap_snp_c2_50176 (**Table 2**), and its single-marker analysis revealed no statistically significant difference when comparing the averages of each genotype group (data not shown). This SNP was located in the middle of the PGSC0003DMG400021635 genome sequence coordinate, which had DNA sequences of a conserved gene, but its function is unknown (Hamilton et al., 2011; Sharma et al., 2013; Uitdewilligen et al., 2013; Spud Database, 2020). Within the 200-kb interval around the solcap_snp_c2_50176 SNP, eight genome sequence coordinates have additionally been observed and are reported in **Supplementary Table 3**. Further research targeting this specific zone is necessary to elucidate the relationship between tuber weight and PGSC0003DMG400021635.

When the QTL map patterns of TW_clo and TW_clo_2019 BLUP datasets were compared with those of the TW_clo_2020 BLUP data, similar delineations were confirmed (**Figure 2**) commonly having the highest peak at 54.06 cM on chromosome 5 even though those highest peaks of TW_clo and TW_clo_2019 BLUP datasets did not reach the LOP significance threshold. As explained above, TW was measured to get tuber size information of the tested tubers indirectly; thus, it is assumed that at least one gene affecting tuber size exists adjacent to 54.06 cM on chromosome 5, and the gene is significantly affected by either environmental or G × E effects. However, more sophisticated examinations for TW_clo_2020_ch05 with a bigger population size and more environmental conditions are needed to examine whether the significant QTL consistently appears across the different environments or not. Also, by collecting total tuber weight data from each plot, it is expected to determine whether the TW_clo_2020_ch05 is also associated with total yield.

## Conclusion

This study mainly conducted the QTL analyses, with a biparental tetraploid mapping population from two russet potatoes, for tuber shape and specific gravity. A previous study conducted by Park et al. (2021) also evaluated a different biparental tetraploid russet mapping population in Aberdeen, ID, USA, in different years. Interestingly, the two studies detected major tuber shape QTL on chromosome 10 in a similar region, which is thought to represent the Ro gene (Masson, 1985; Van Eck et al., 1994; Endelman and Jansky, 2016; Chen et al., 2019). On chromosome 4, both studies found significant tuber shape QTL, but QTL were not in close proximity to each other, representing at least two unrelated genes. Nonetheless, chromosome 4, like chromosome 10, appears to impact tuber shape. Park et al. (2021) exclusively reported a tuber shape-related QTL on chromosome 7, and this current study also exclusively identified another QTL impacting tuber shape on chromosome 6. New QTL data were also presented in this study regarding tuber depth, or flatness, which was associated with a region of chromosome 2. During the QTL analyses for specific gravity in this study, a significant QTL was found on chromosome 3, with Park et al. (2021) reporting on SG QTL on chromosomes 1 and 5, as well. Additionally, significant QTL for TW on chromosome 5 was also reported in this study.

The results of this study provide more insights into the genetics of the russet market class with chromosome 10 (Ro gene) being identified as a major contributor to tuber shape across russet mapping populations. The finding, with long tuber shape, not round, is important in the russet market class. Chromosome 4 also appears to contribute to tuber shape. Of interest to processors and the potato industry is the degree of tuber depth (flatness), which can impact marketability. This study, to the best of our knowledge, is the first to report on a significant QTL for tuber depth on chromosome 2. These QTL results can be used for developing markers that can be used in MAS in the russet market class.

Finally, when comparing the performance of the two most commonly used tuber shape measurement methods (LW ratio; quantitative vs. visual assessment; objective), no significant difference was observed in evaluating tuber length from round to long. In particular, both methods showed almost the same performance in localizing the major tuber shape QTL on chromosome 10, thereby commonly proving their reliabilities. However, the visual assessment phenotype data additionally revealed a new significant QTL on chromosome 4 that was not discovered with LW ratio data. This result reflects that when selecting or studying potatoes based on tuber shape, potato breeders or researchers should carefully choose an appropriate measurement method depending on their main purpose.

## Data availability statement

The original contributions presented in the study are included in the article/**Supplementary Material**. Further inquiries can be directed to the corresponding author.

## Author contributions

JP: Conceptualization, Data curation, Formal Analysis, Investigation, Methodology, Project administration, Software, Supervision, Writing – original draft, Writing – review & editing, Resources, Validation, Visualization. JW: Conceptualization, Funding acquisition, Project administration, Resources, Supervision, Validation, Writing – review & editing. RN: Conceptualization, Data curation, Funding acquisition, Investigation, Methodology, Project administration, Resources, Supervision, Validation, Writing – review & editing.
