# Supplementary figures and images for: QTL identified that influence tuber length–width ratio, degree of flatness, tuber size, and specific gravity in a russet-skinned, tetraploid mapping population
Source: Front Plant Sci. 2024 Mar 22;15:1343632. doi: 10.3389/fpls.2024.1343632 (PMC10996053; doi:10.3389/fpls.2024.1343632)

**Supplementary Figure 1. Measurement methods for tuber length, width, and depth**


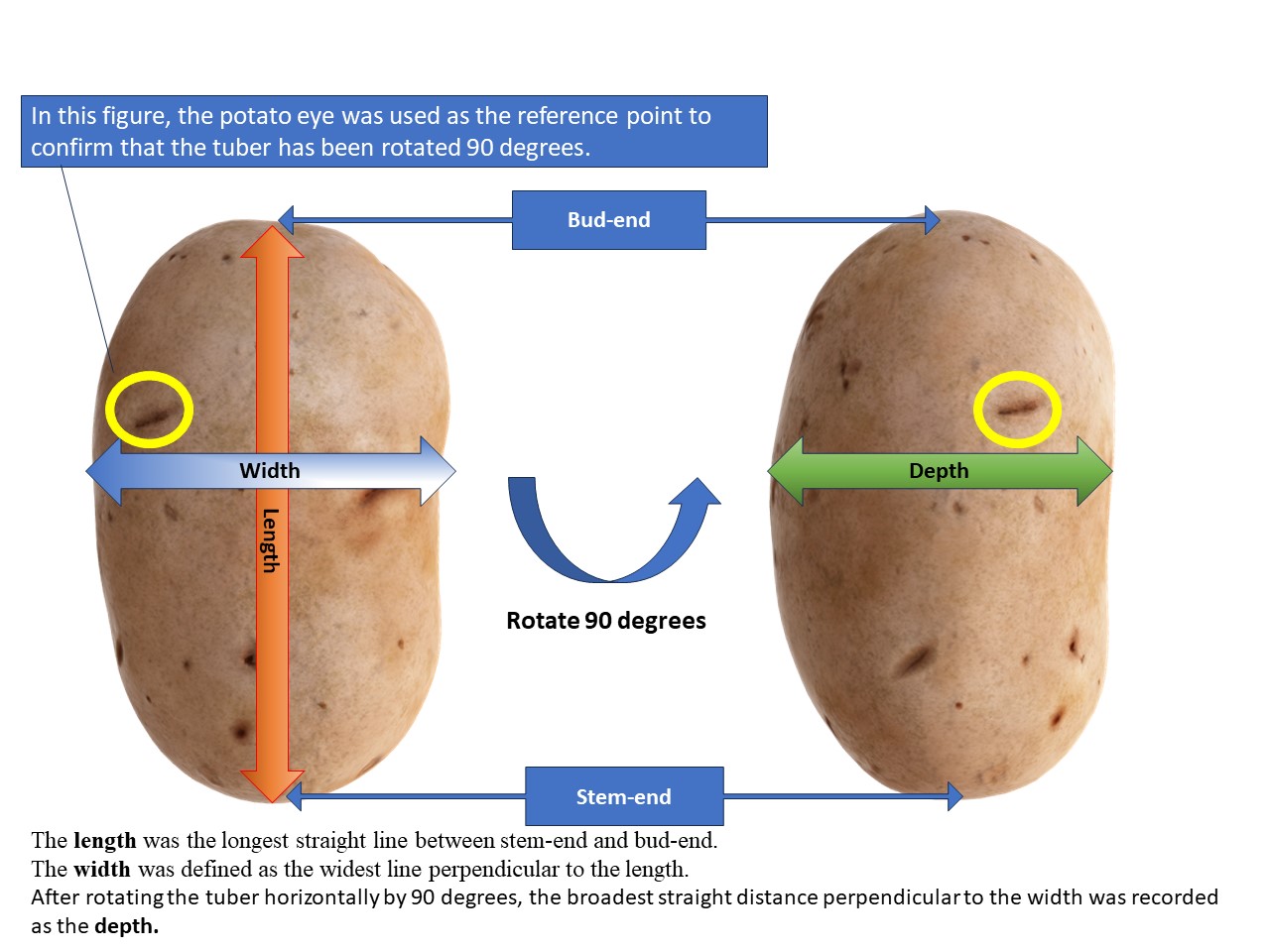

Supplement: Supplementary file 1 [file DataSheet_1.docx]

**Supplementary Figure 2. Scale for tuber shape measurement (SolCAP 2009)**


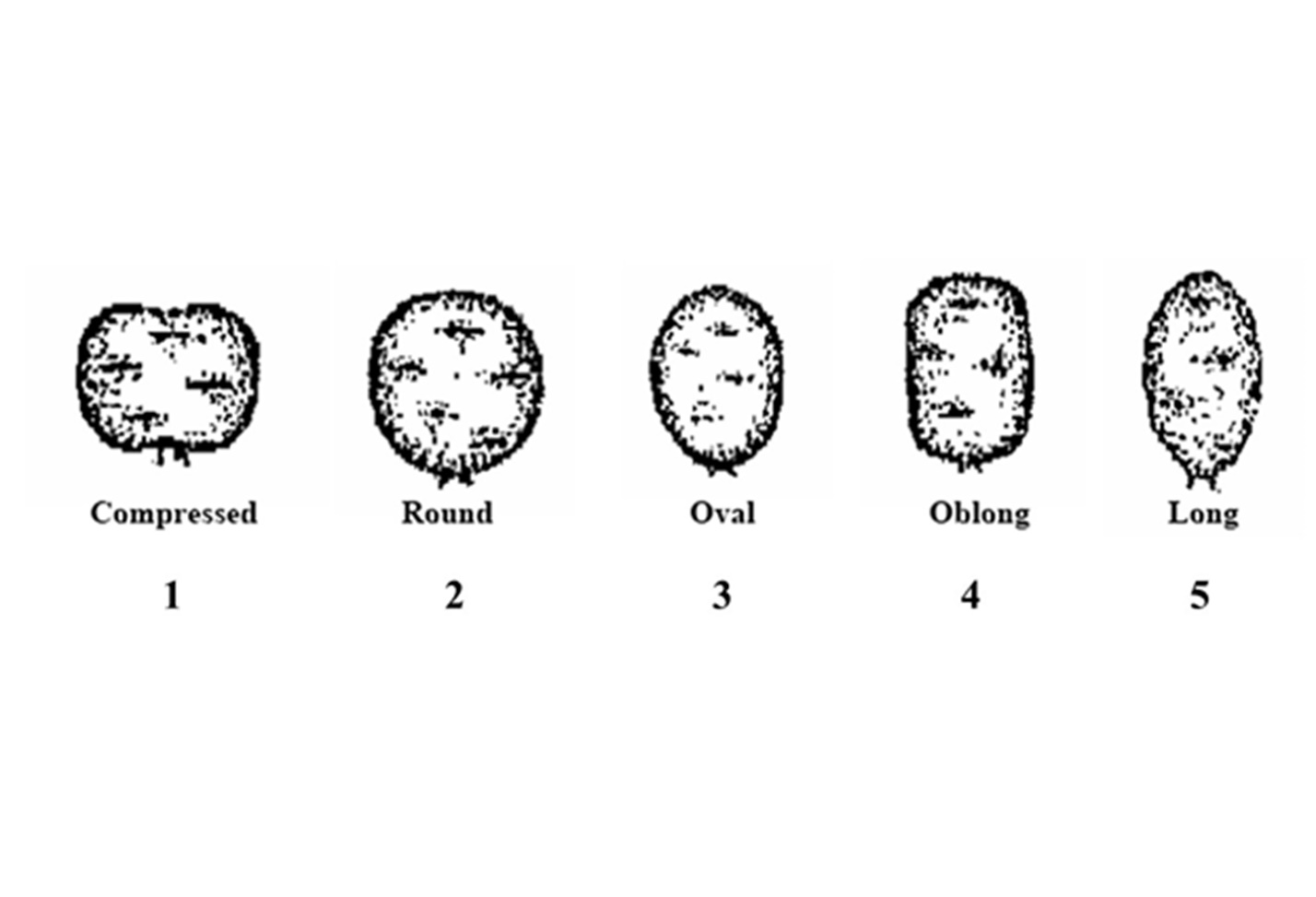

Supplement: Supplementary file 2 [file DataSheet_2.docx]
