# Supplementary material for: QTL identified that influence tuber length–width ratio, degree of flatness, tuber size, and specific gravity in a russet-skinned, tetraploid mapping population
Source: Front Plant Sci. 2024 Mar 22;15:1343632. doi: 10.3389/fpls.2024.1343632 (PMC10996053; doi:10.3389/fpls.2024.1343632)
Supplement: Supplementary file 3 [file DataSheet_3.docx]

**Supplementary Figure 3. Distribution** **of the BLUP datasets of length-width ratio (LW), width-depth ratio (WD), tuber shape visual assessment (VA), specific gravity (SG), and tuber weight (TW)**

**
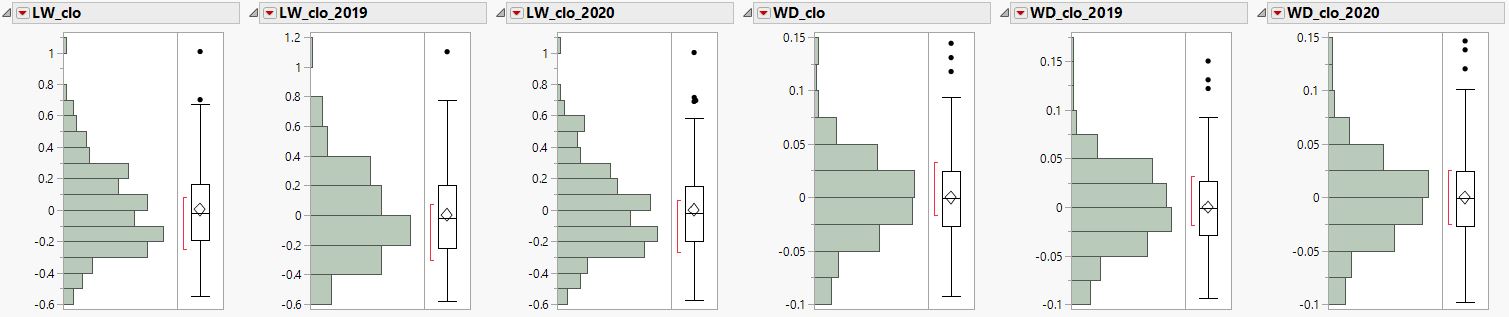
**

**
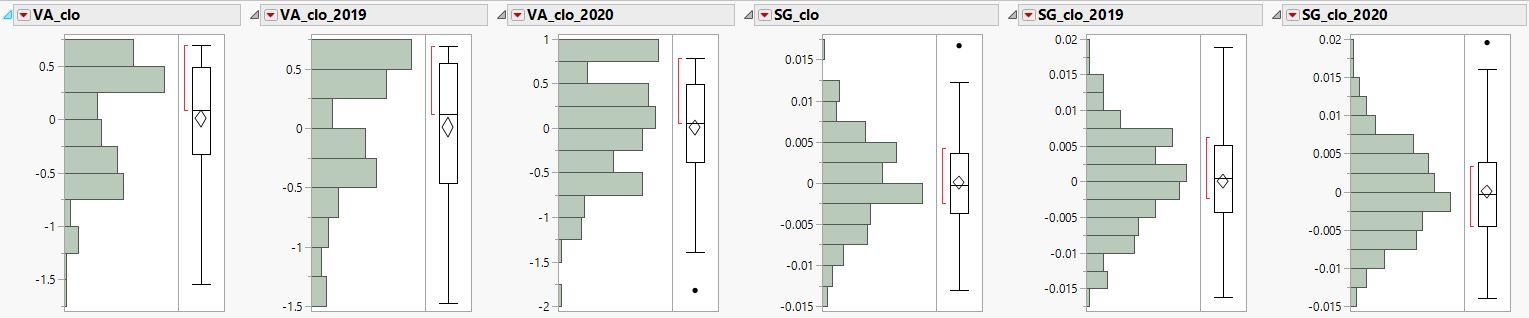
**

**
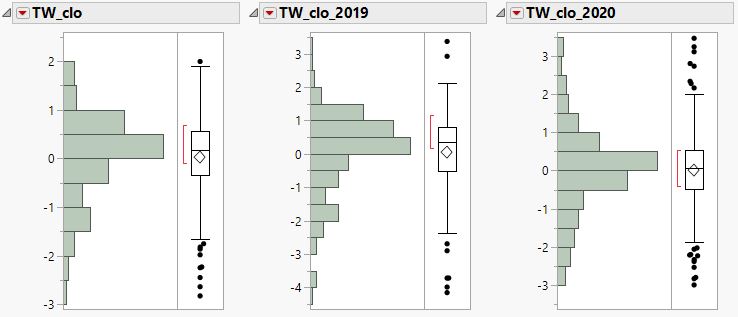
**
