# Supplementary material for: QTL identified that influence tuber length–width ratio, degree of flatness, tuber size, and specific gravity in a russet-skinned, tetraploid mapping population
Source: Front Plant Sci. 2024 Mar 22;15:1343632. doi: 10.3389/fpls.2024.1343632 (PMC10996053; doi:10.3389/fpls.2024.1343632)

Supplementary Figure 4. Allele effects of the significant QTL positions

- The "a" to "d" at the X axes in the following bar graphs represent four phased homologs of Palisade Russet, and the "e" to "h" represent another four homologs of ND028673B-2Russ. The Y axes in the same bar graphs indicate each homolog's contribution to the average of the whole mapping population. The *qtl_effects* function of QTLpoly calculated an estimate of each allele effect across the eight homologs; thus, all the Y axes in Supplementary Figure 4 were labeled as “Estimate” without any unit. Through those bar graphs, it was possible to quantify how much each homolog of each parent adds to or subtracts from the mean of the 190 progenies, revealing which allele(s) among the eight parental homologs most significantly impact a trait.
- BLUP data abbreviations: Length-Width ratio (LW), Width-Depth ratio (WD), Tuber shape Visual Assessment (VA), Specific Gravity (SG), Tuber Weight (TW), a genetic effect of clones (clo), 2019 (2019), and 2020 (2020) year effects

LW_clo_ch10 SNP (Location): solcap_snp_c2_25471 (40.05 cM)
Chromosome: chr10
BLUP data: *LW_clo*

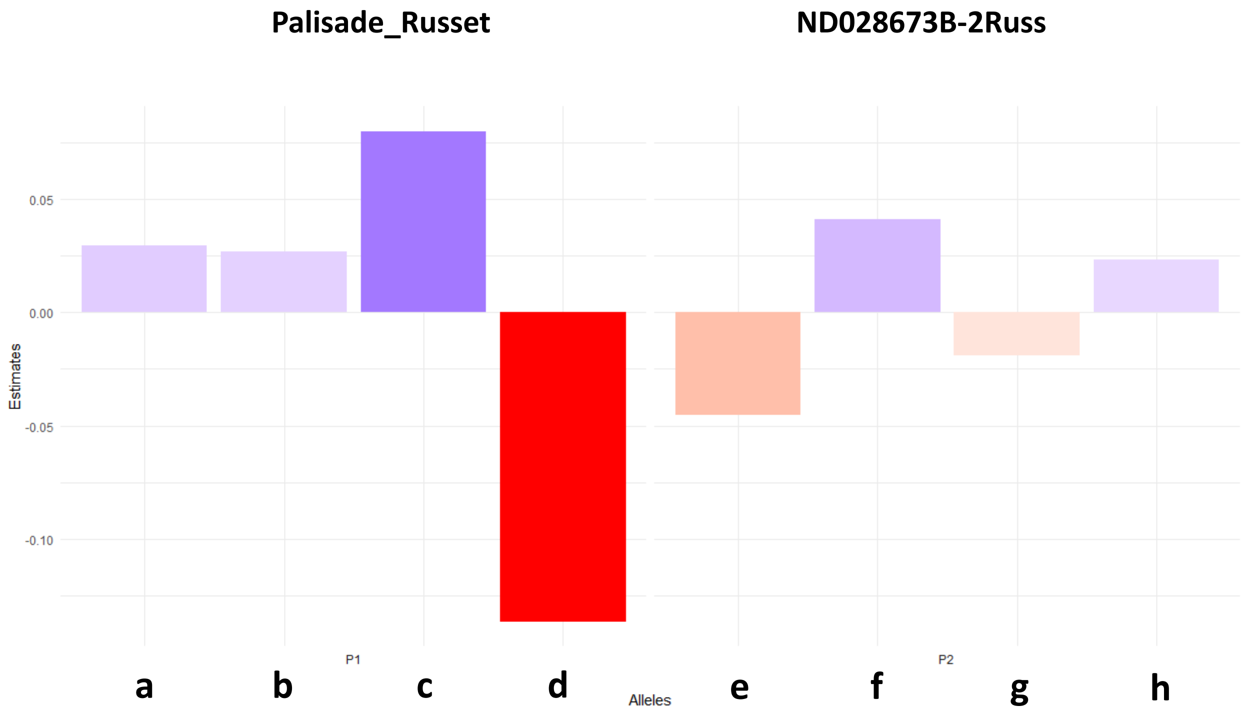

LW_clo_2019_ch10 SNP (Location): solcap_snp_c2_25471 (40.05 cM)
Chromosome: chr10
BLUP data: *LW_clo_2019*

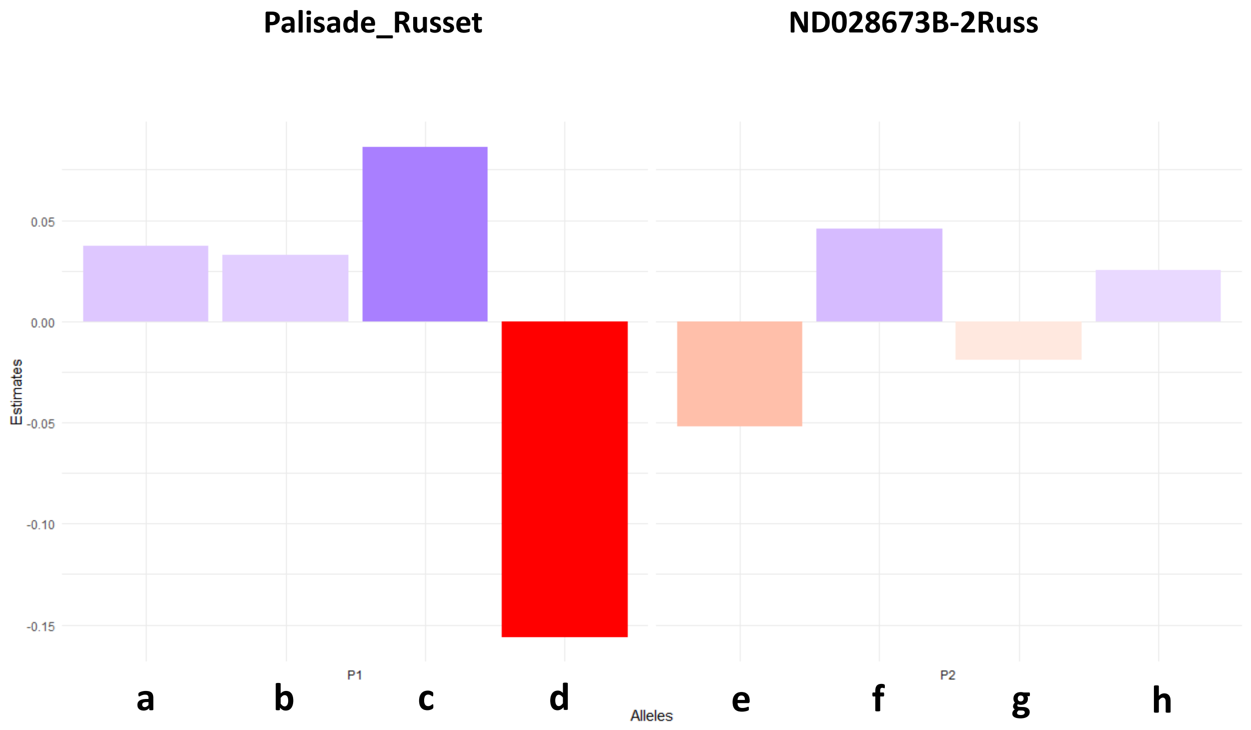

LW_clo_2020_ch10 SNP (Location): solcap_snp_c2_25471 (40.05 cM)
Chromosome: chr10
BLUP data: *LW_clo_2020*

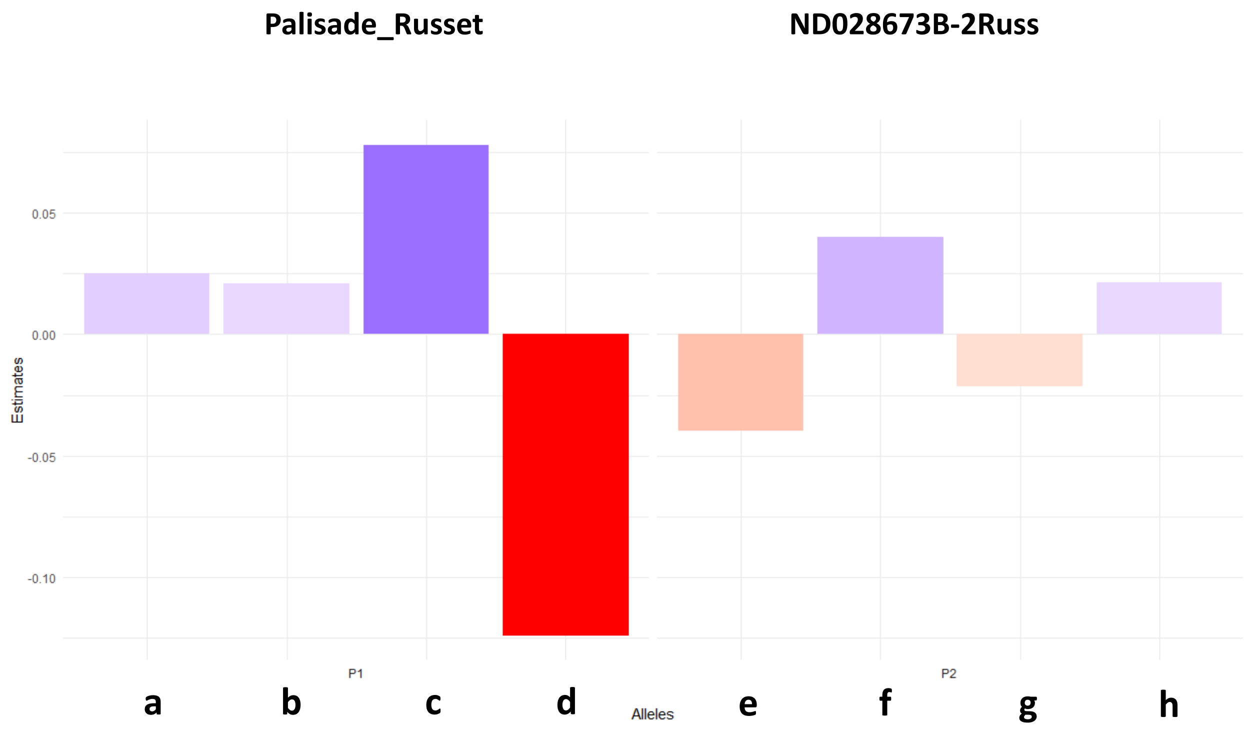

LW_clo_2020_ch06

SNP (Location): solcap_snp_c2_31648 (35.44 cM)
Chromosome: chr06
BLUP data: *LW_clo_2020*

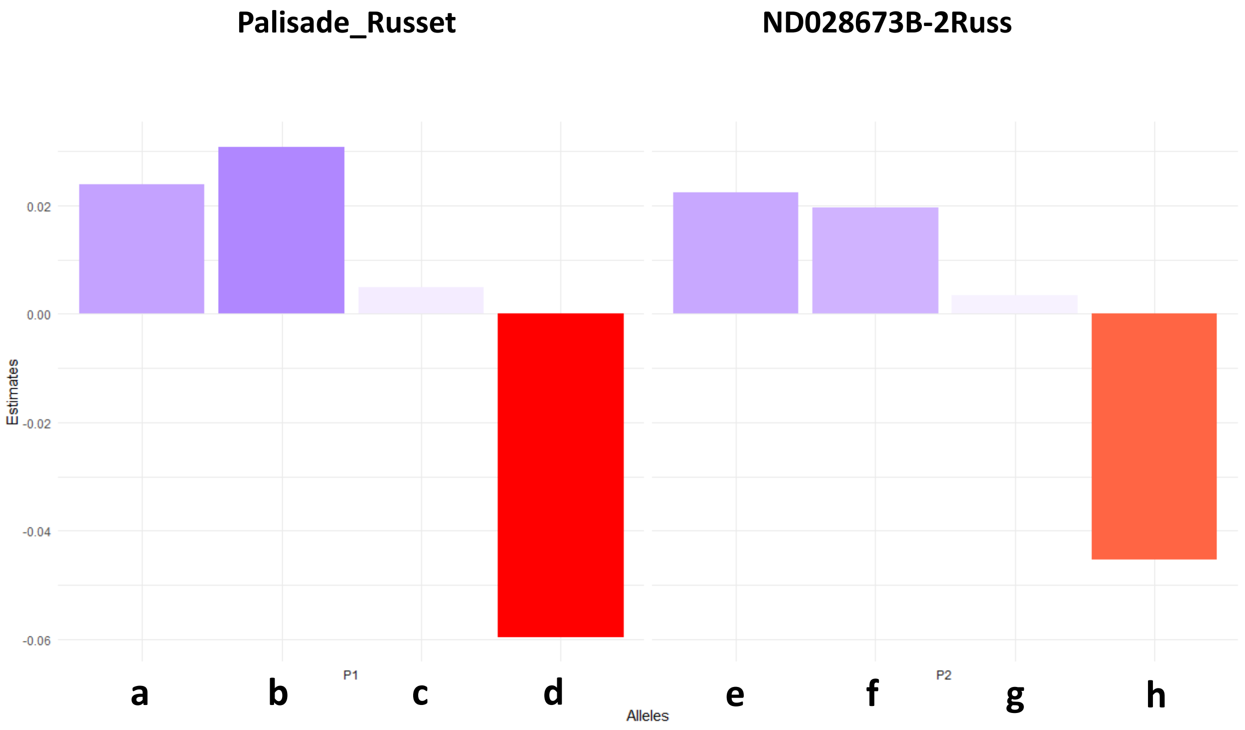

_VA_clo_ch04_ SNP (Location): PotVar0075244 (74.04 cM)
Chromosome: chr04
BLUP data: *VA_clo*

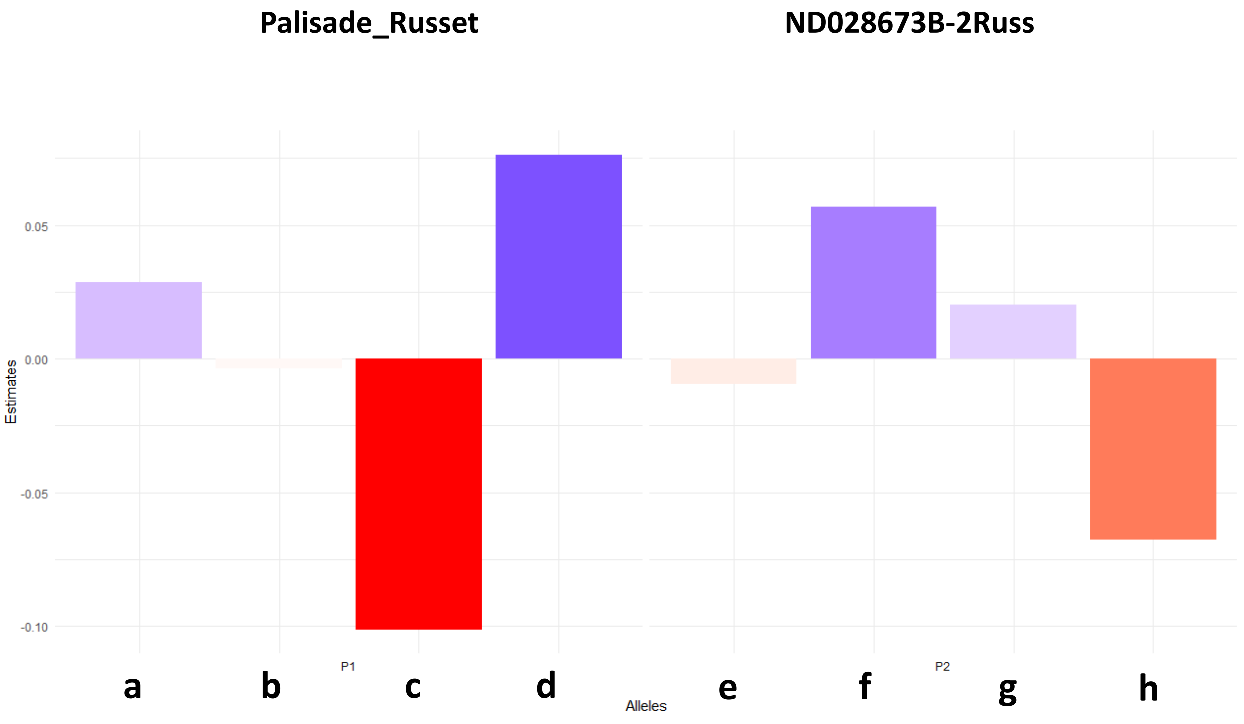

_VA_clo_2019_ch04_ SNP (Location): PotVar0075244 (74.04 cM)
Chromosome: chr04
BLUP data: *VA_clo_2019*

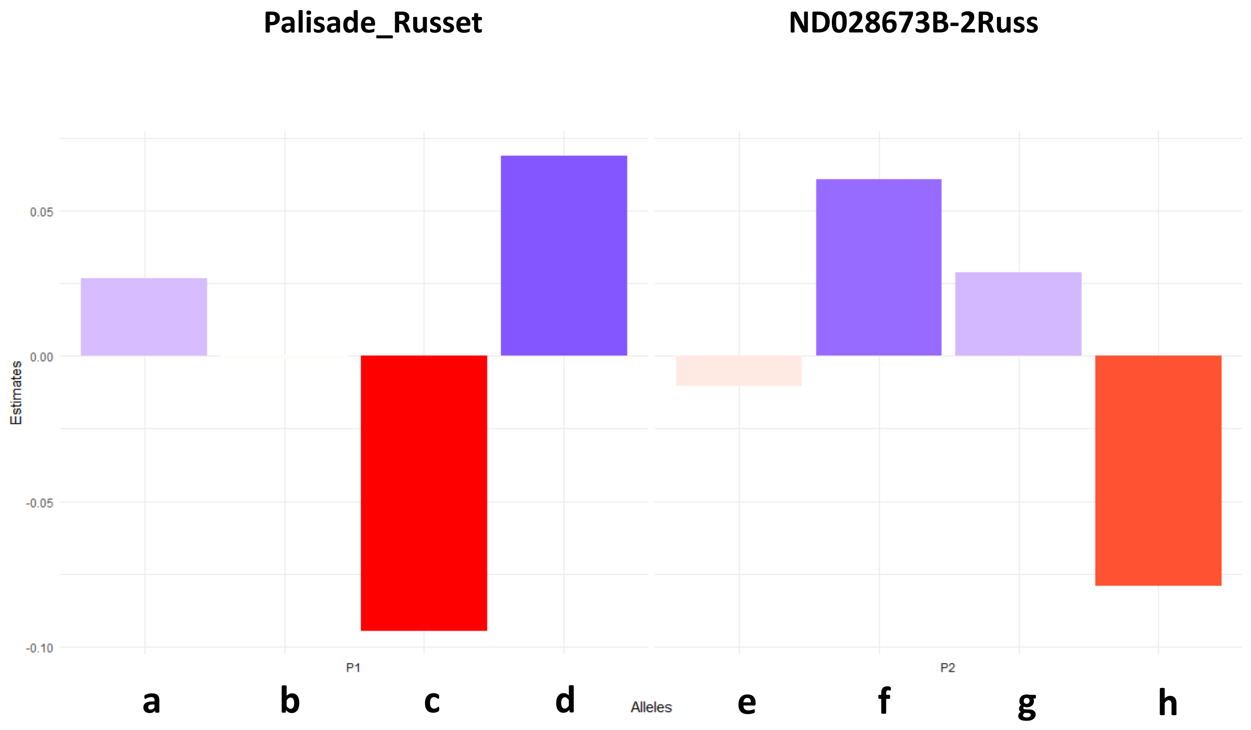

_VA_clo_2020_ch04_ SNP (Location): PotVar0075244 (74.04 cM)
Chromosome: chr04
BLUP data: *VA_clo_2020*

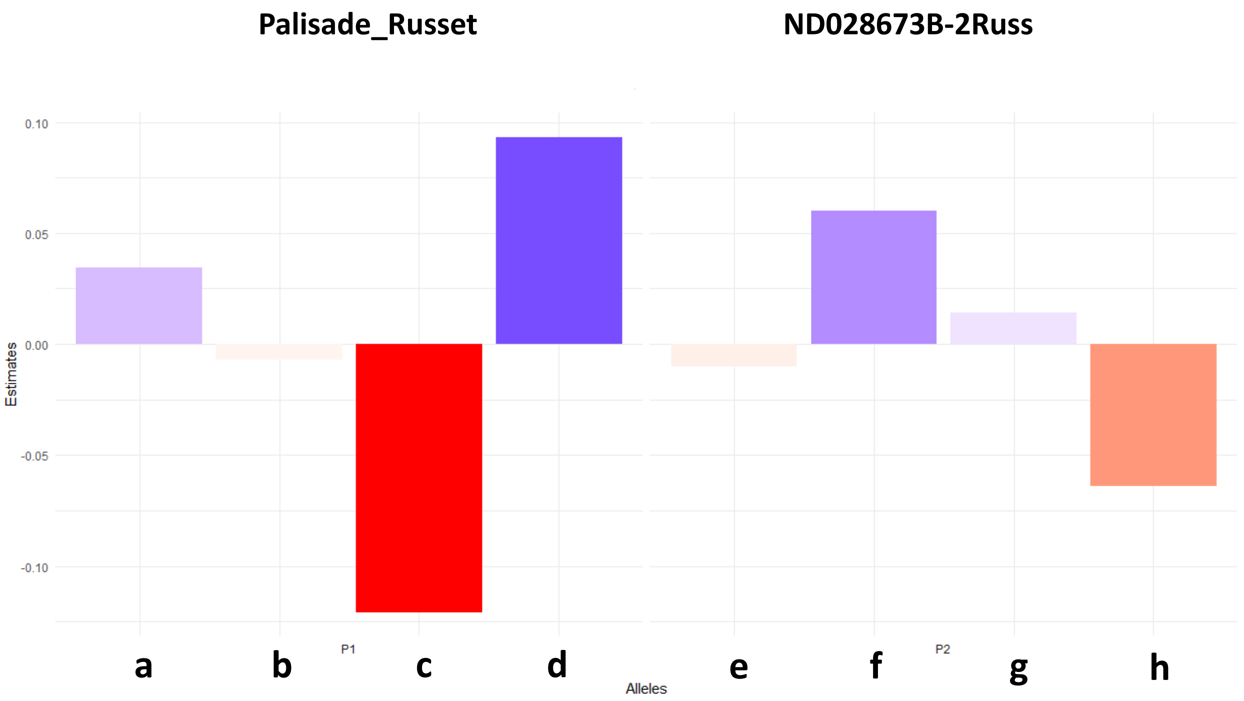

VA_clo_ch10

SNP (Location): solcap_snp_c2_25471 (40.05 cM)
Chromosome: chr10
BLUP data: *VA_clo*

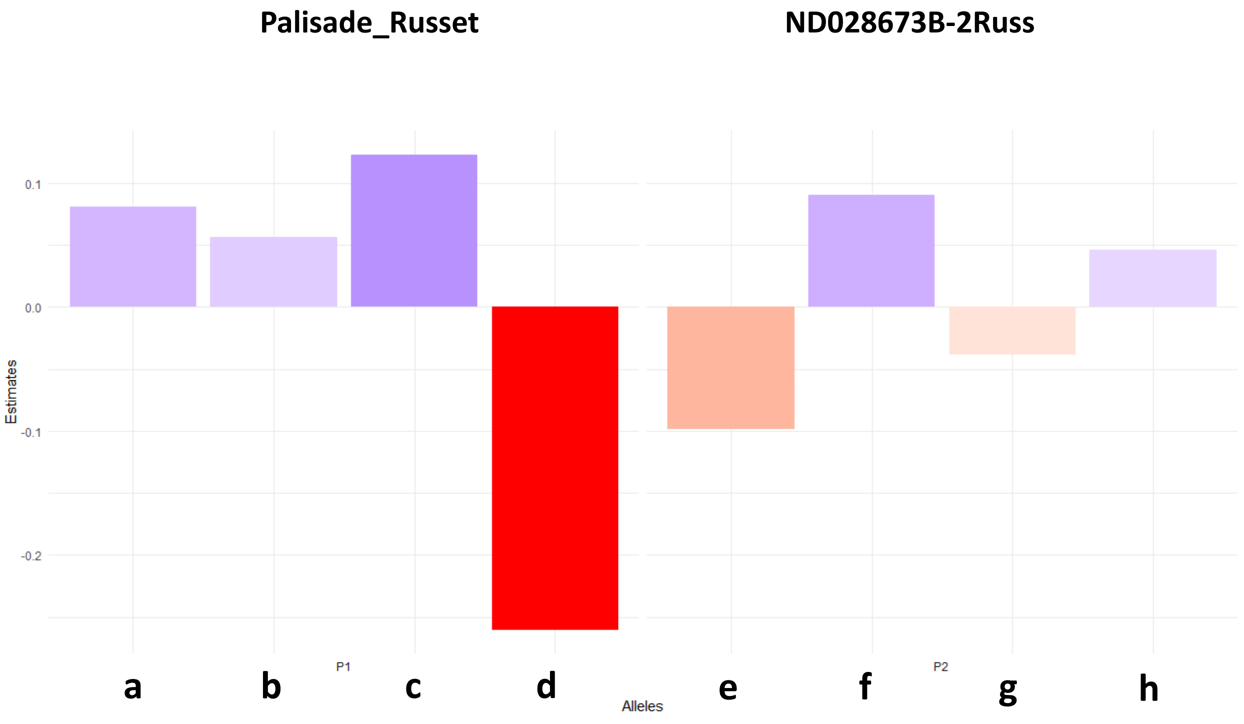

VA_clo_2019_ch10

SNP (Location): solcap_snp_c2_25471 (40.05 cM)
Chromosome: chr10
BLUP data: *VA_clo_2019*

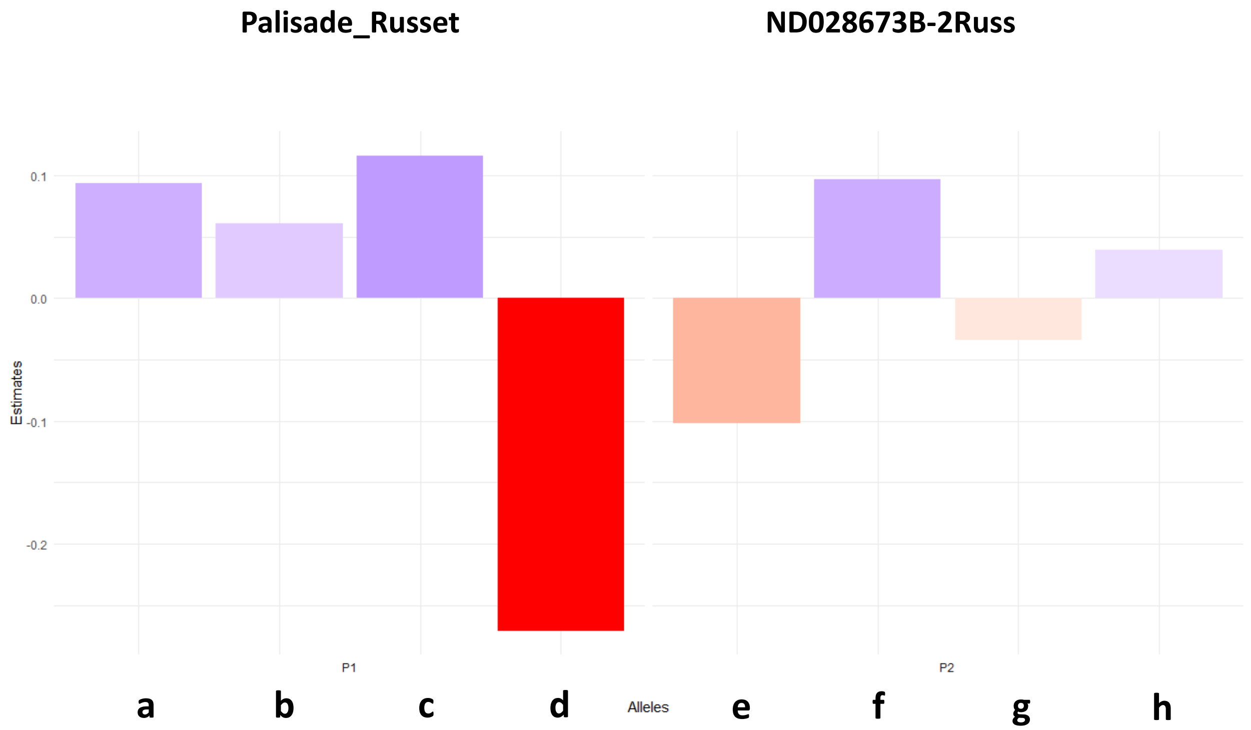

VA_clo_2020_ch10

SNP (Location): solcap_snp_c2_25471 (40.05 cM)
Chromosome: chr10
BLUP data: *LW_clo_2020*

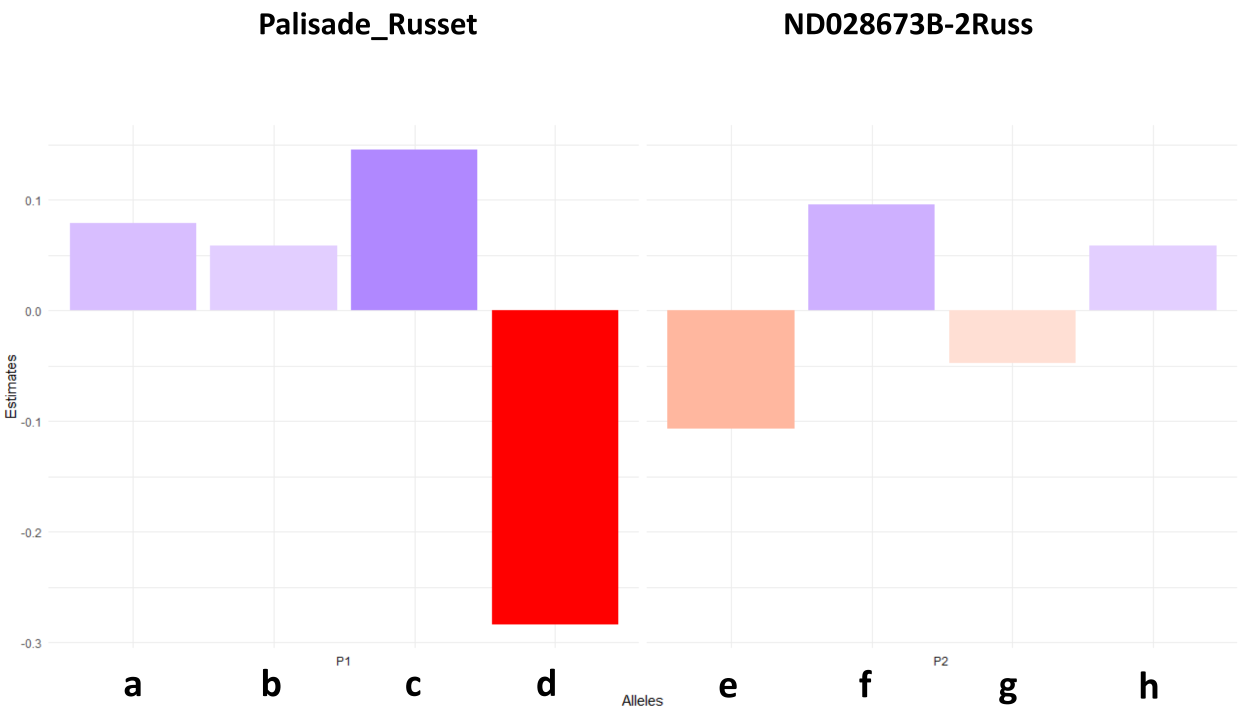

WD_clo_ch02 SNP (Location): solcap_snp_c2_41980 (29.20 cM)
Chromosome: chr02
BLUP data: *WD_clo*

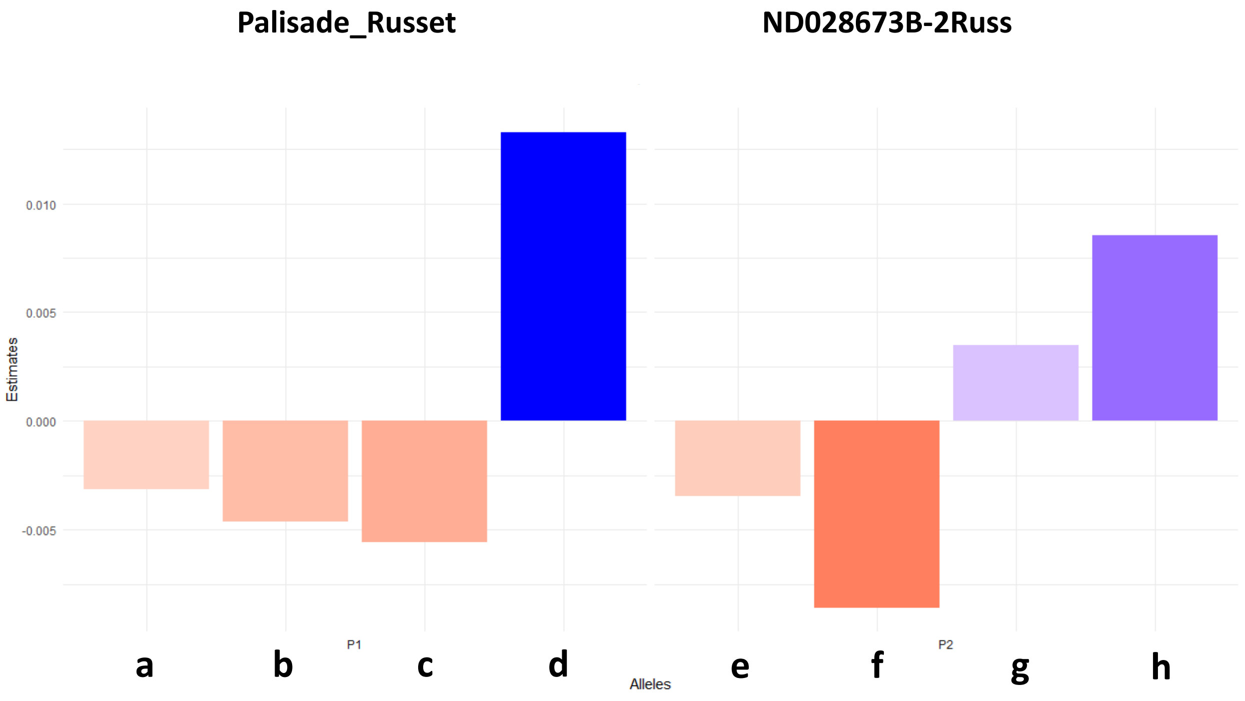

WD_clo_2019_ch02 SNP (Location): solcap_snp_c2_41980 (29.20 cM)
Chromosome: chr02
BLUP data: *WD_clo_2019*

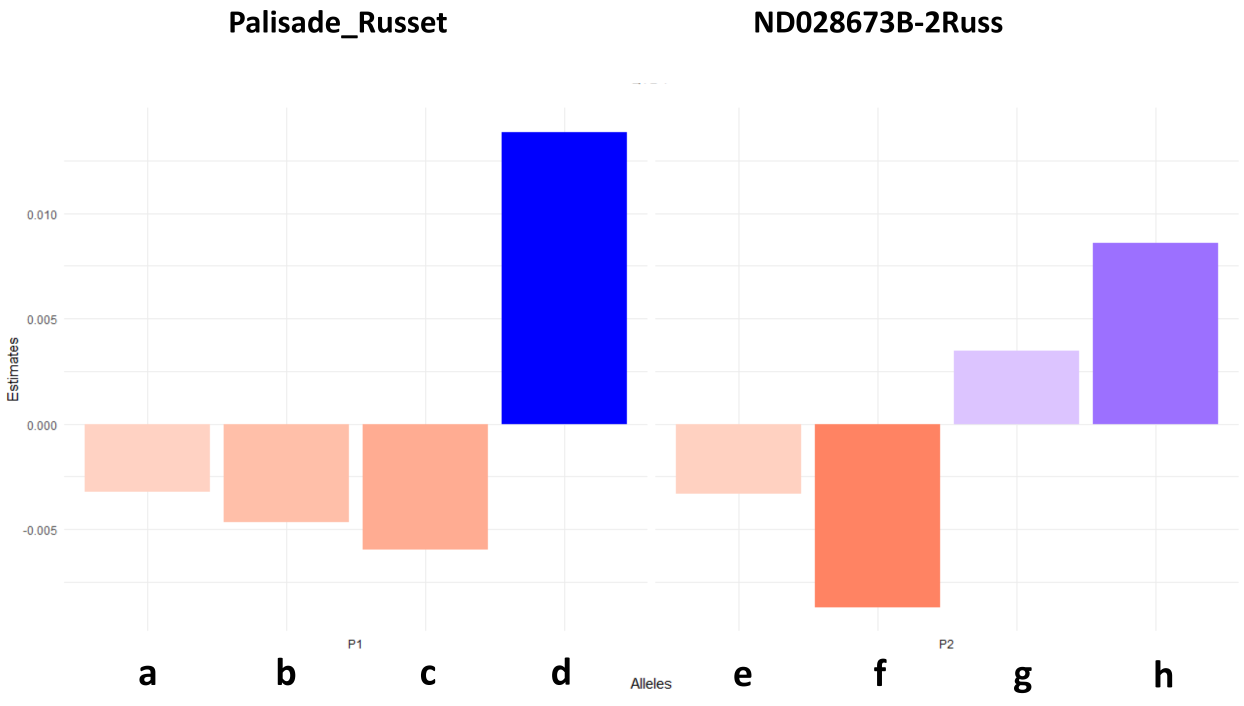

WD_clo_2020_ch02 SNP (Location): solcap_snp_c2_41980 (29.20 cM)
Chromosome: chr02
BLUP data: *WD_clo_2020*

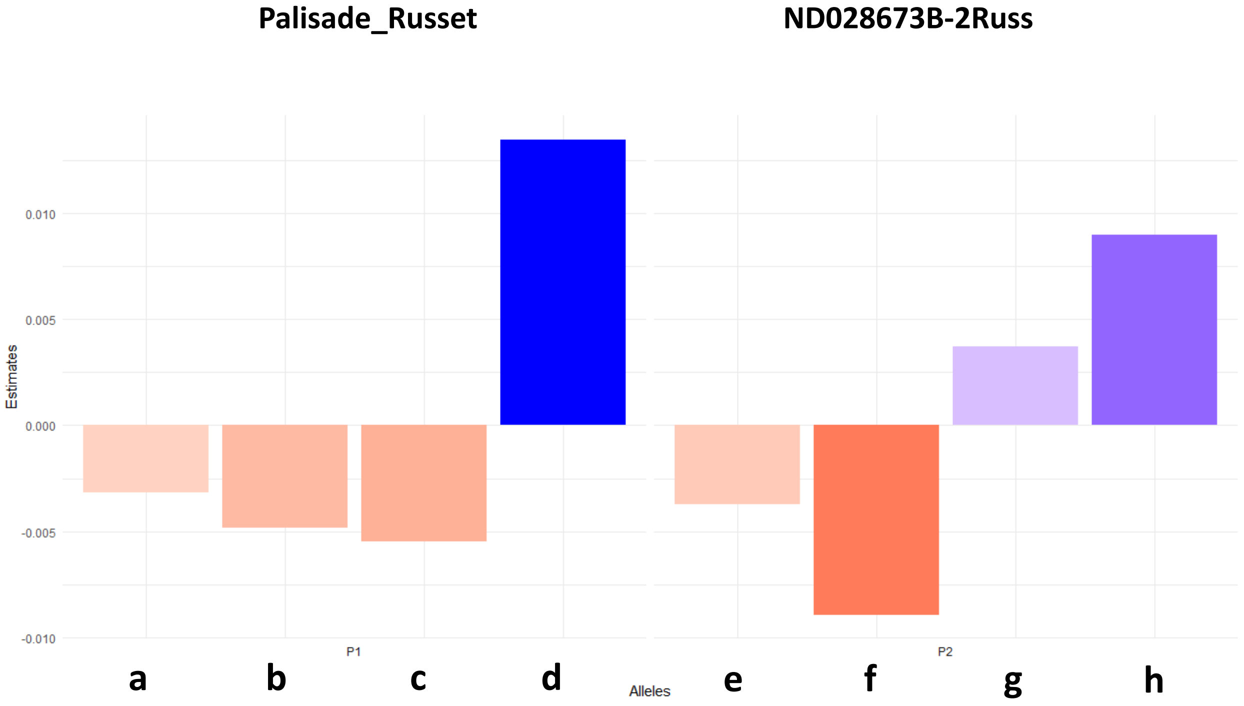

SG_clo_ch03

SNP (Location): solcap_snp_c1_3348 (17.05 cM)
Chromosome: chr03
BLUP data: *SG_clo*

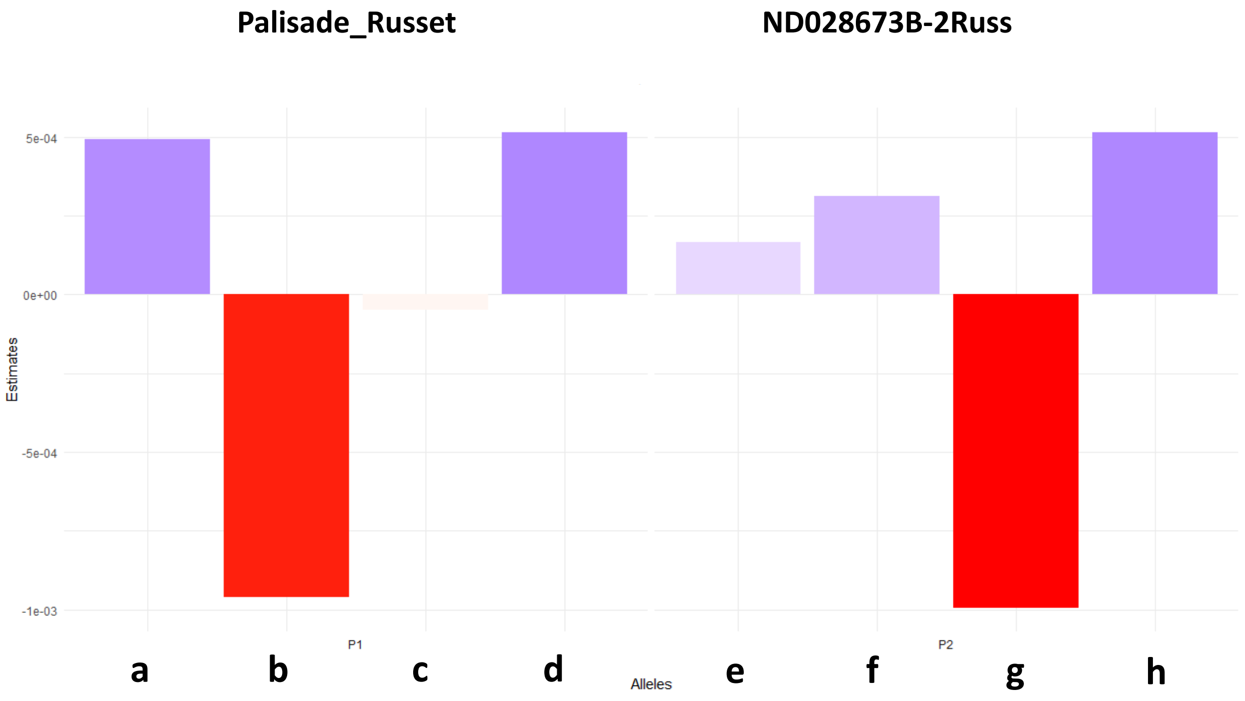

SG_clo_2020_ch03

SNP (Location): solcap_snp_c1_3348 (17.05 cM)
Chromosome: chr03
BLUP data: SG_clo_2020

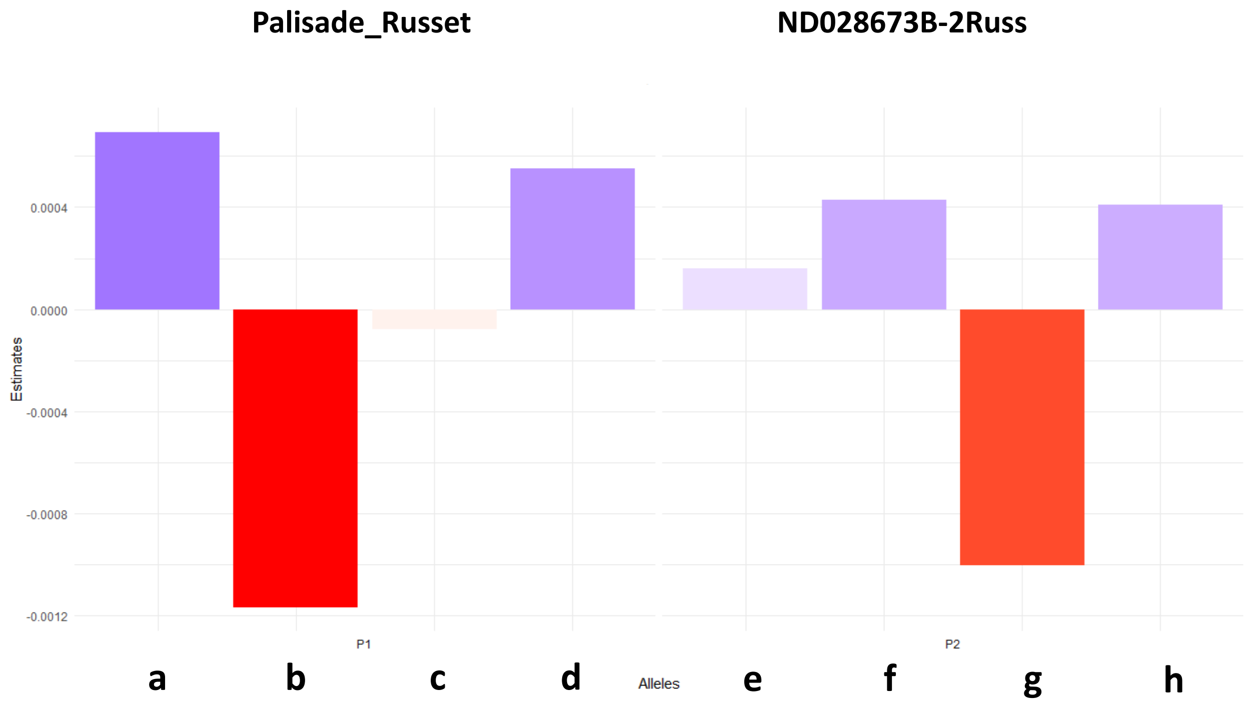

TW_clo_2020_ch05

SNP (Location): solcap_snp_c2_50176 (54.06 cM)
Chromosome: chr05
BLUP data: TW_clo_2020

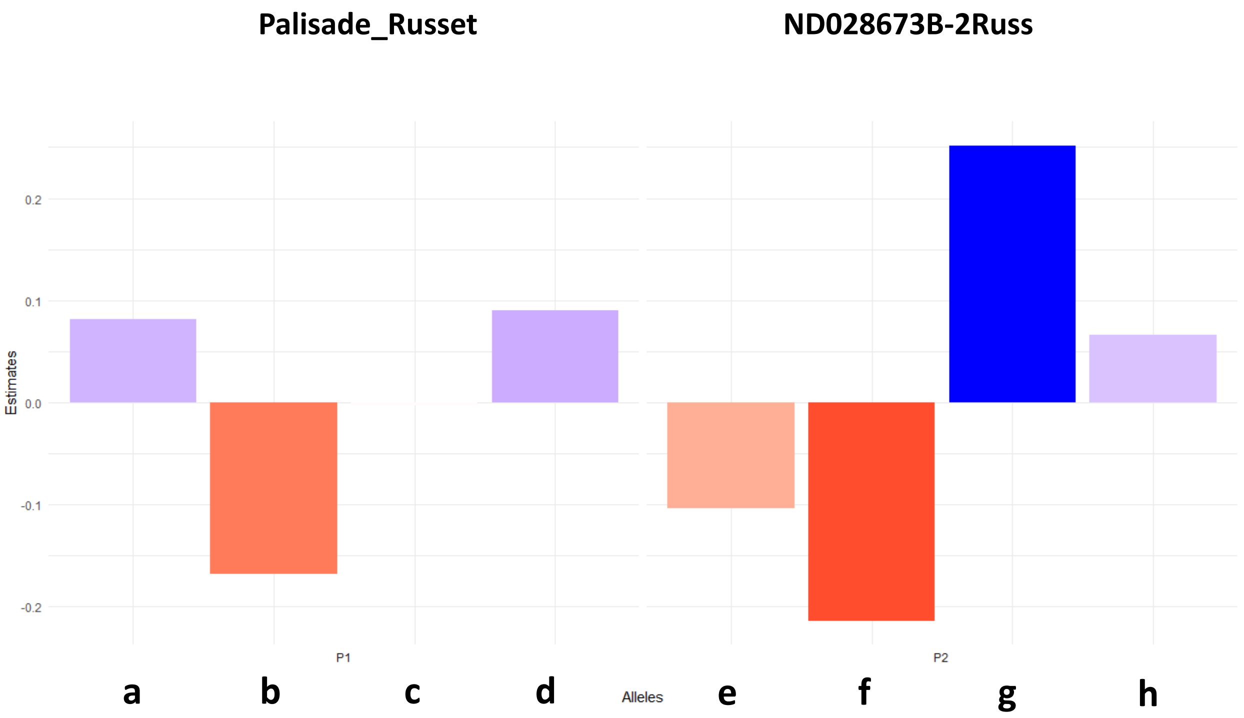

Supplement: Supplementary file 4 [file DataSheet_4.pdf]
