# Supplementary material for: QTL identified that influence tuber length–width ratio, degree of flatness, tuber size, and specific gravity in a russet-skinned, tetraploid mapping population
Source: Front Plant Sci. 2024 Mar 22;15:1343632. doi: 10.3389/fpls.2024.1343632 (PMC10996053; doi:10.3389/fpls.2024.1343632)
Supplement: Supplementary file 5 [file DataSheet_5.pdf]

Supplementary Figure 5. Single-marker analyses for the SNPs linked to LW, VA, WD, & SG QTL

a_1 to _6. Single-marker analyses for SNPs linked to LW QTL

b_1 to _6. Single-marker analyses for SNPs linked to VA QTL

c. Single-marker analyses for SNP linked to WD QTL

d_1 to _2. Single-marker analyses for SNPs linked to SG QTL

The Tukey-Kramer mean comparison test was employed in the single-marker analysis with a significance threshold of p-value < 0.05. Significance was indicated under the group heading using letters "X" and "Y" when the p-value was below 0.05, denoting that the two groups were significantly different. For instance, if the genotypic groups are significantly different, they will be labeled as X and Y, respectively. In cases where a genotype group is not significantly different from another, it will be designated as "XY." The distinctions are also represented visually through circles; for example, if two circles do not overlap, it signifies that the two means are significantly different from each other. Each green diamond shape displayed on the BLUP segregation pattern of each genotype group represents the group mean and the range of its standard error.

a_1

"solcap_snp_c2_25471" Single-Marker analysis

Mean comparison test (Tukey-Kramer; p-value < 0.05)

LW_clo_ch10

LW_clo

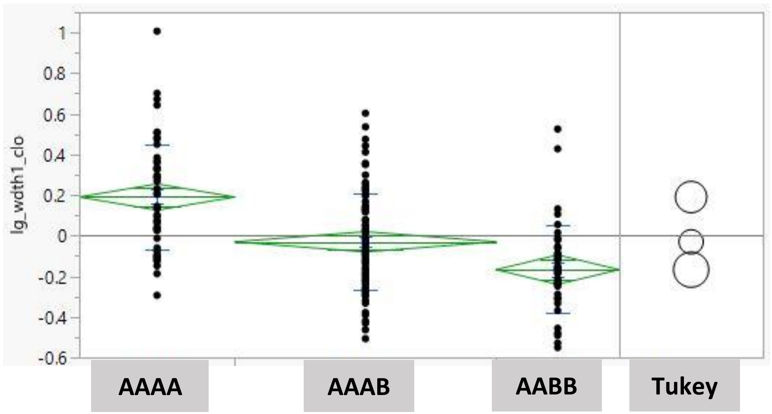

Genotypes	Freq.	Mean	Group
AAAA	53	0.19055	X
AAAB	89	-0.03005	Y
AABB	42	-0.16733	Z

LW_clo_2019_ch10

LW_clo_2019

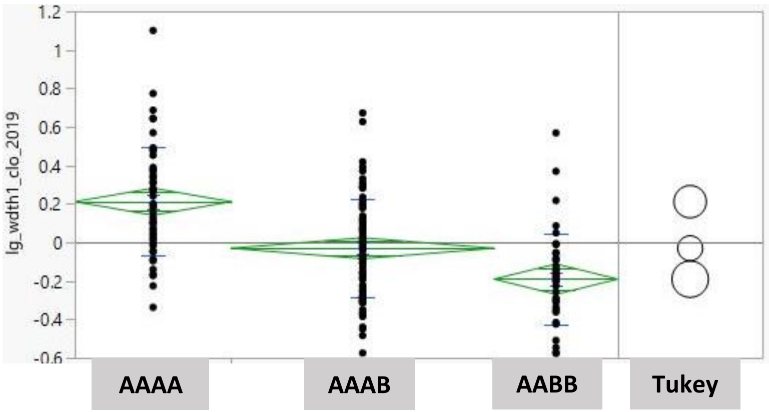

Genotypes	Freq.	Mean	Group
AAAA	53	0.211652	X
AAAB	89	-0.03016	Y
AABB	42	-0.19075	Z

LW_clo_2020_ch10

LW_clo_2020

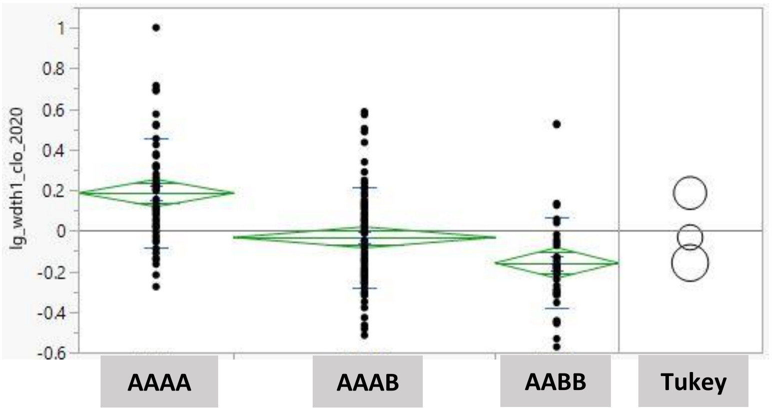

Genotypes	Freq.	Mean	Group
AAAA	53	0.185976	X
AAAB	89	-0.03254	Y
AABB	42	-0.15843	Z

a_2

"solcap_snp_c2_25469" Single-Marker analysis

Mean comparison test (Tukey-Kramer; *p*-value < 0.05)

LW_clo_ch10

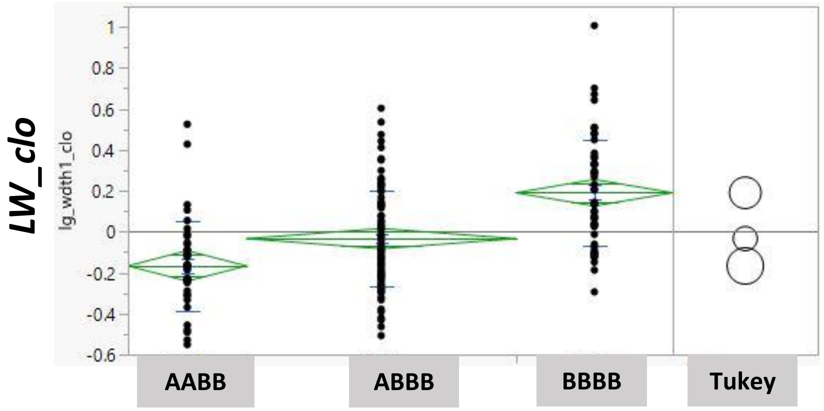

Genotypes	Freq.	Mean	Group
AABB	40	-0.16669	X
ABBB	91	-0.03335	Y
BBBB	53	0.19055	Z

LW_clo_2019_ch10

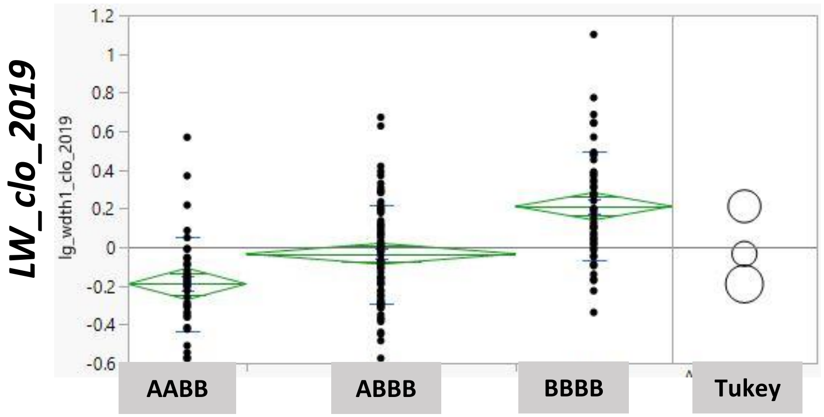

Genotypes	Freq.	Mean	Group
AABB	40	-0.19002	X
ABBB	91	-0.03401	Y
BBBB	53	0.21165	Z

LW_clo_2020_ch10

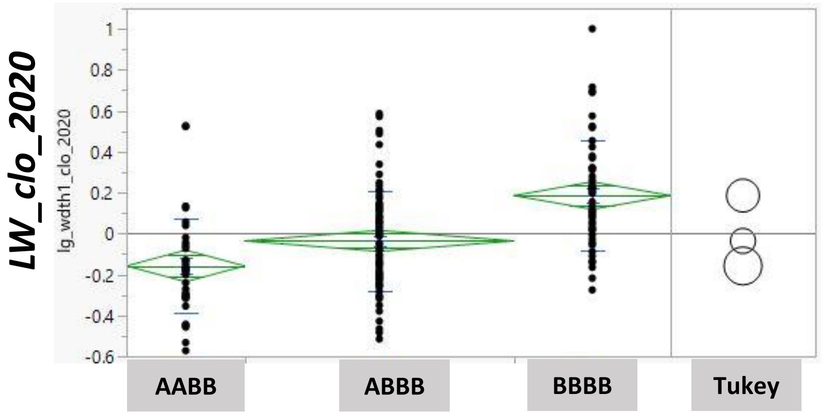

Genotypes	Freq.	Mean	Group
AABB	40	-0.15781	X
ABBB	91	-0.03558	Y
BBBB	53	0.185976	Z

a_3

“solcap_snp_c1_8021” Single-Marker analysis

Mean comparison test (Tukey-Kramer; *p*-value < 0.05)

LW_clo_ch10

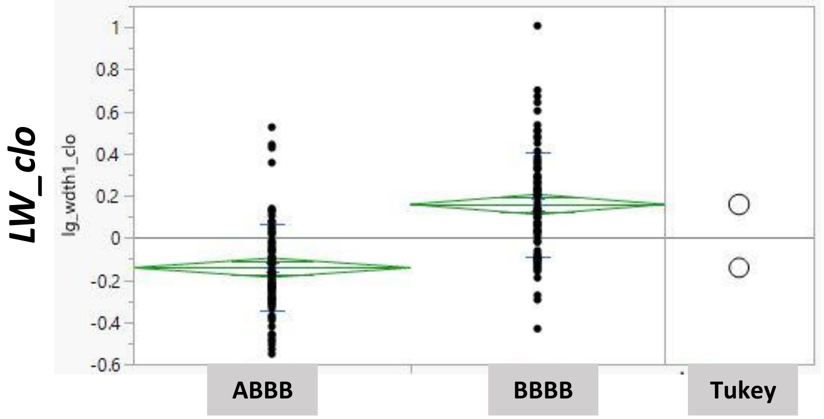

Genotypes	Freq.	Mean	Group
ABBB	96	-0.14145	X
BBBB	88	0.15882	Y

LW_clo_2019_ch10

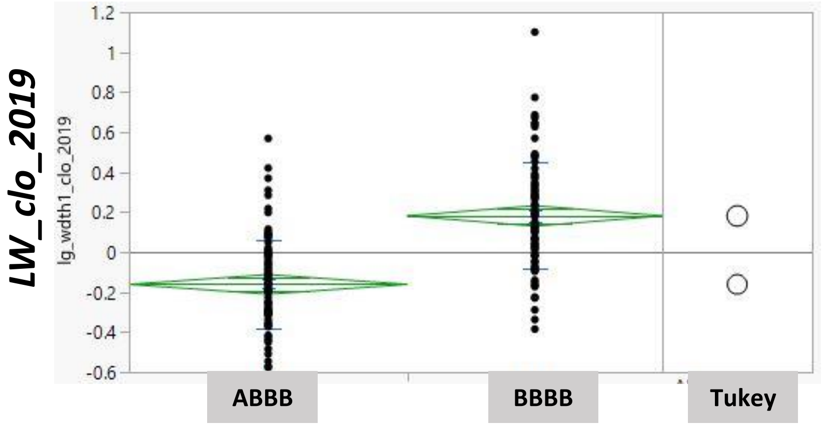

Genotypes	Freq.	Mean	Group
ABBB	96	-0.16087	X
BBBB	88	0.181419	Y

LW_clo_2020_ch10

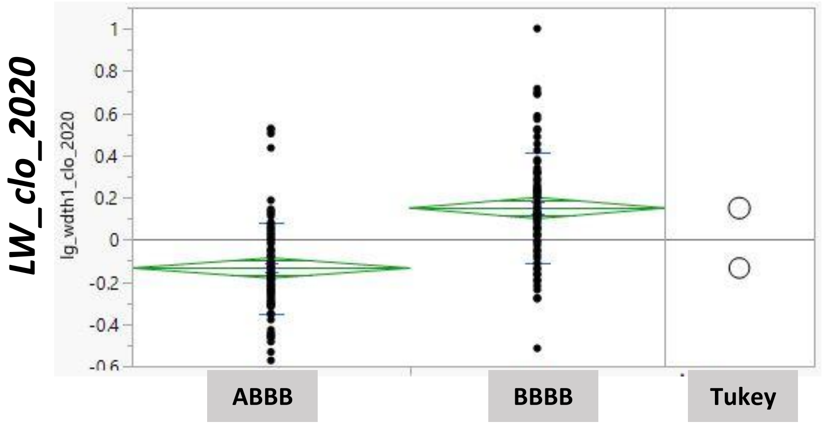

Genotypes	Freq.	Mean	Group
ABBB	96	-0.13431	X
BBBB	88	0.15	Y

a_4

"solcap_snp_c1_8020" Single-Marker analysis

LW_clo_ch10

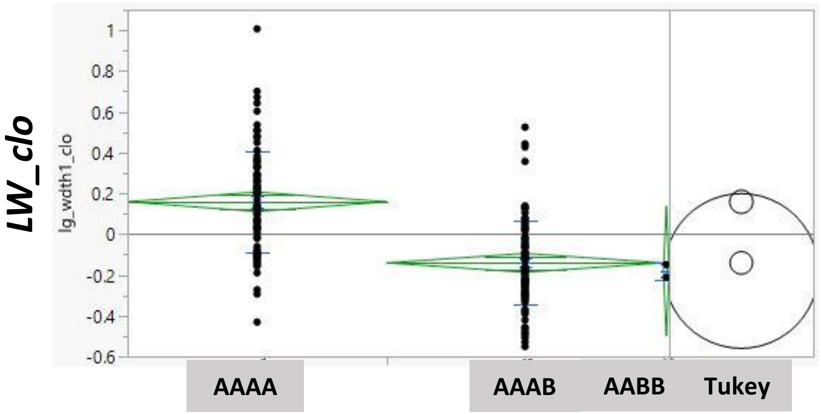

LW_clo_2019_ch10

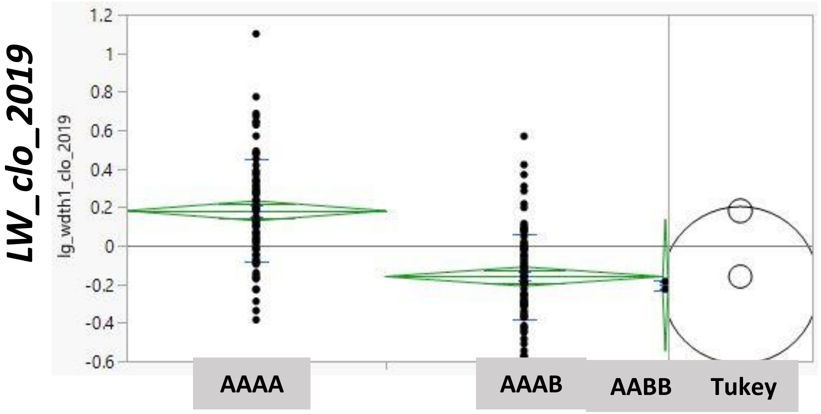

LW_clo_2020_ch10

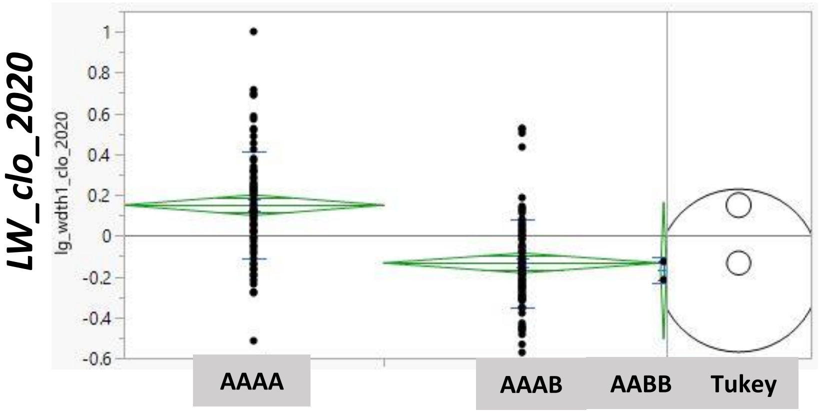

Mean comparison test (Tukey-Kramer; *p*-value < 0.05)

Genotypes	Freq.	Mean	Group
AAAA	88	0.15882	X
AAAB	94	-0.14063	Y
AABB	2	-0.1803	XY

Genotypes	Freq.	Mean	Group
AAAA	88	0.181419	X
AAAB	94	-0.15992	Y
AABB	2	-0.20553	XY

Genotypes	Freq.	Mean	Group
AAAA	88	0.15	X
AAAB	94	-0.13353	Y
AABB	2	-0.17071	XY

a_5

"solcap_snp_c1_8019" Single-Marker analysis

Mean comparison test (Tukey-Kramer; *p*-value < 0.05)

LW_clo_ch10

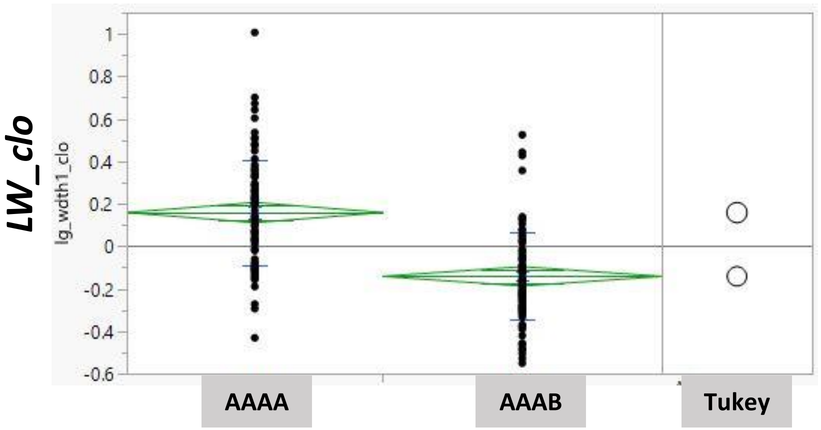

Genotypes	Freq.	Mean	Group
AAAA	88	0.15882	X
AAAB	96	-0.14145	Y

LW_clo_2019_ch10

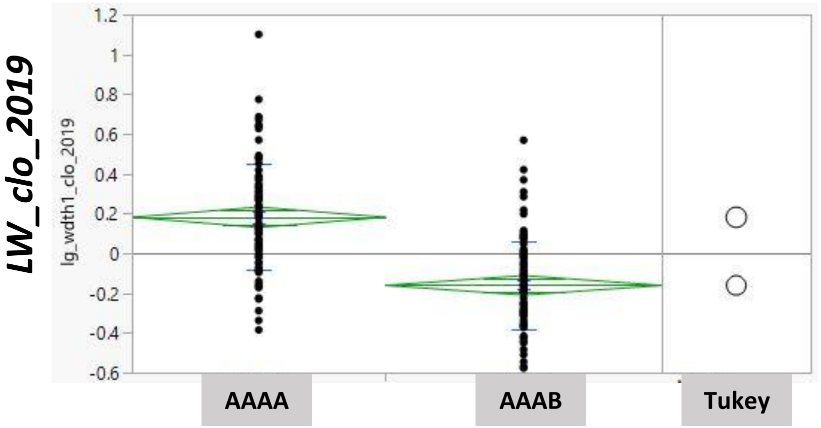

Genotypes	Freq.	Mean	Group
AAAA	88	0.18142	X
AAAB	96	-0.16087	Y

LW_clo_2020_ch10

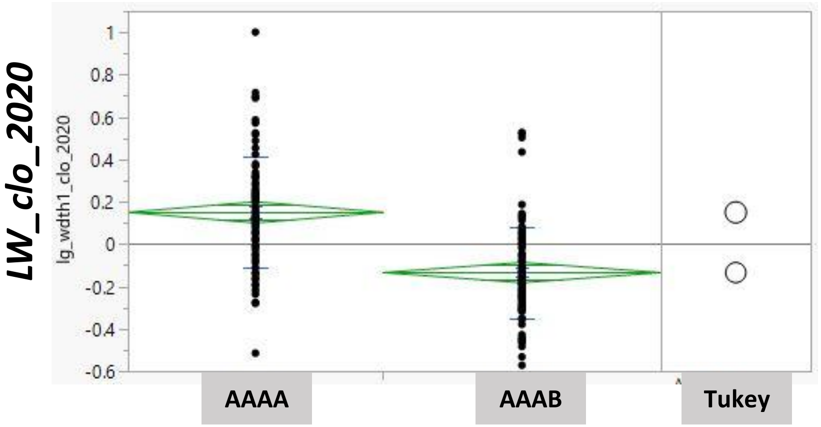

Genotypes	Freq.	Mean	Group
AAAA	88	0.15	X
AAAB	96	-0.13431	Y

a_6

"solcap_snp_c2_31648" Single-Marker analysis

LW_clo_2020_ch06

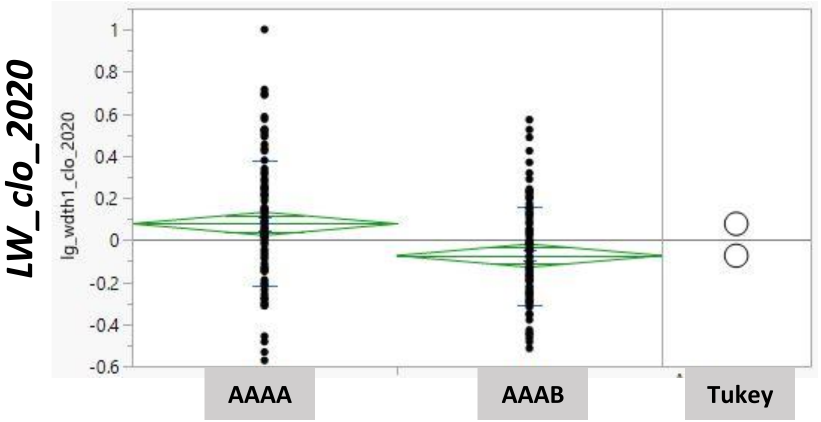

Mean comparison test (Tukey-Kramer; *p*-value < 0.05)

Genotypes	Freq.	Mean	Group
AAAA	92	0.07737	X
AAAB	92	-0.07404	Y

b_1

“solcap_snp_c2_25471” Single-Marker analysis

VA_clo_ch10

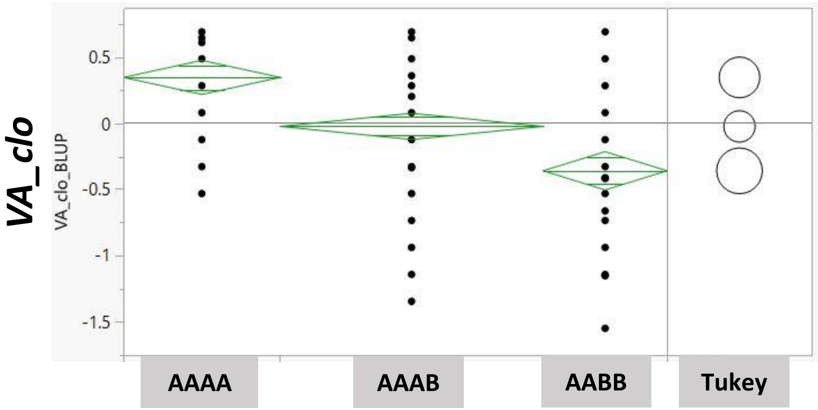

VA_clo_2019_ch10

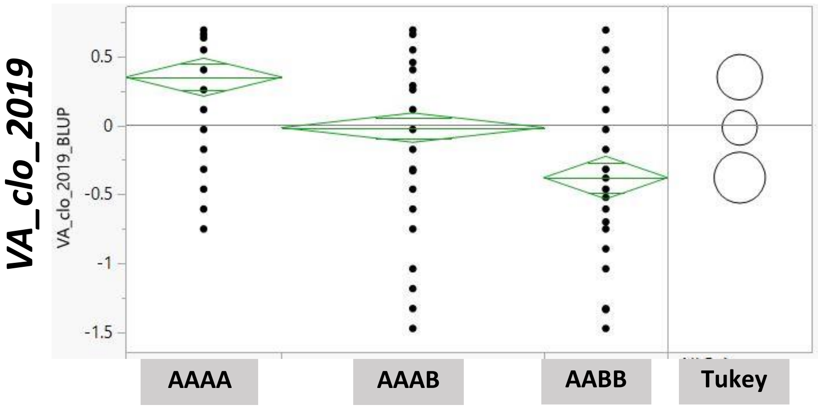

L VA _clo_2020_ch10

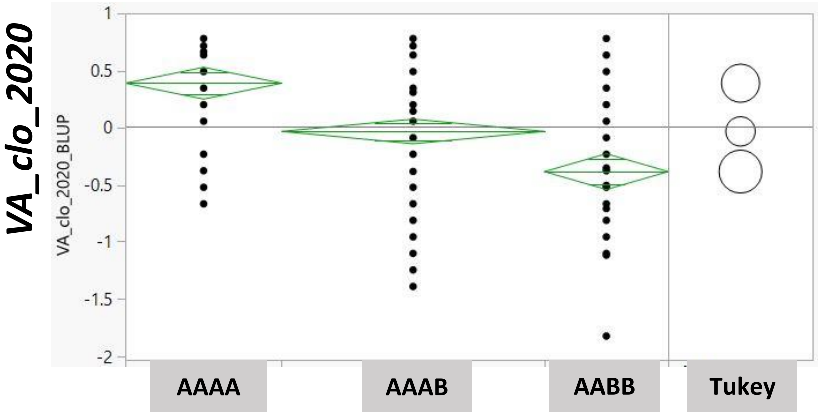

Mean comparison test (Tukey-Kramer; *p*-value < 0.05)

Genotypes	Freq.	Mean	Group
AAAA	53	0.34624	X
AAAB	89	-0.0233	Y
AABB	42	-0.35814	Z

Genotypes	Freq.	Mean	Group
AAAA	53	0.34948	X
AAAB	89	-0.01715	Y
AABB	42	-0.38004	Z

Genotypes	Freq.	Mean	Group
AAAA	53	0.38901	X
AAAB	89	-0.03254	Y
AABB	42	-0.38382	Z

b_2

“solcap_snp_c2_25469” Single-Marker analysis

VA_clo_ch10

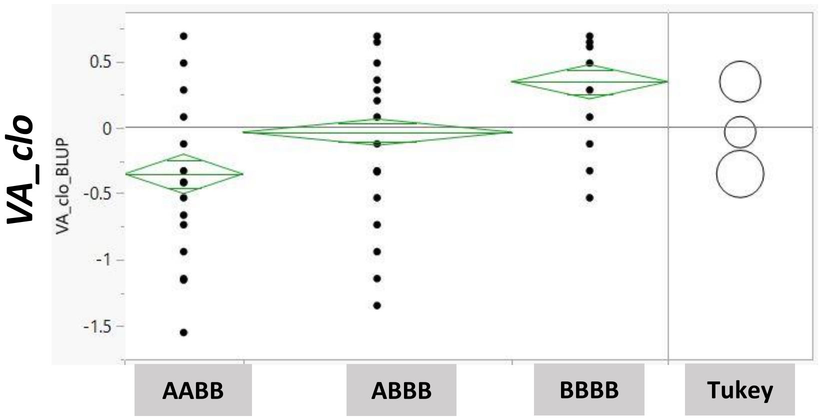

VA_clo_2019_ch10

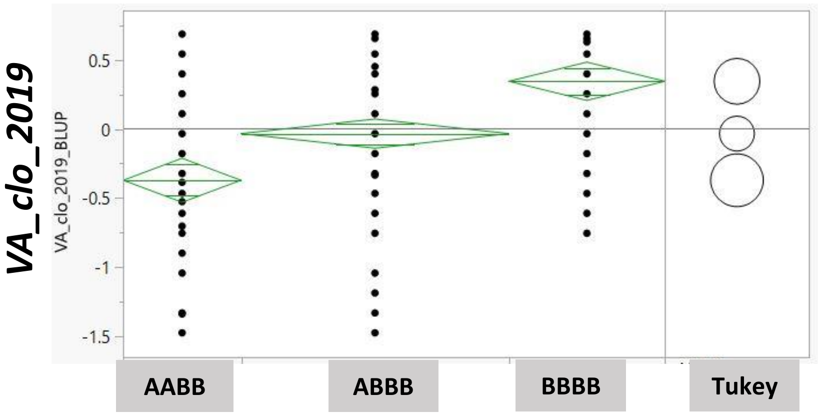

VA_clo_2020_ch10

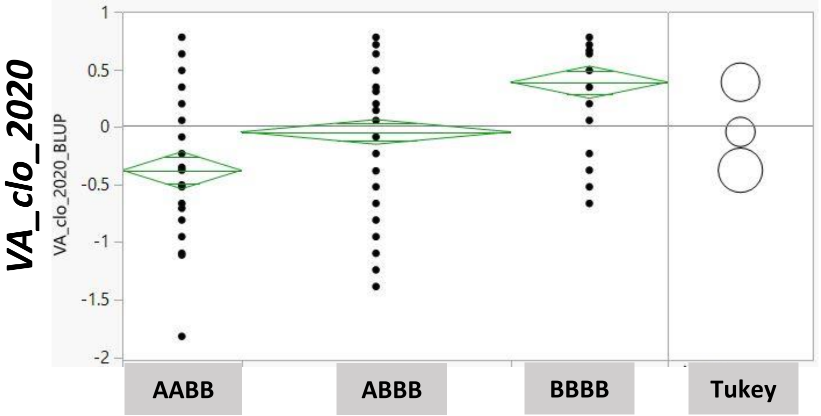

Mean comparison test (Tukey-Kramer; *p*-value < 0.05)

Genotypes	Freq.	Mean	Group
AABB	40	-0.34956	X
ABBB	91	-0.03443	Y
BBBB	53	0.34624	Z

Genotypes	Freq.	Mean	Group
AABB	40	-0.36861	X
ABBB	91	-0.03015	Y
BBBB	53	0.34948	Z

Genotypes	Freq.	Mean	Group
AABB	40	-0.37696	X
ABBB	91	-0.04328	Y
BBBB	53	0.38901	Z

b_3

“solcap_snp_c1_8021” Single-Marker analysis

Mean comparison test (Tukey-Kramer; *p*-value < 0.05)

VA_clo_ch10

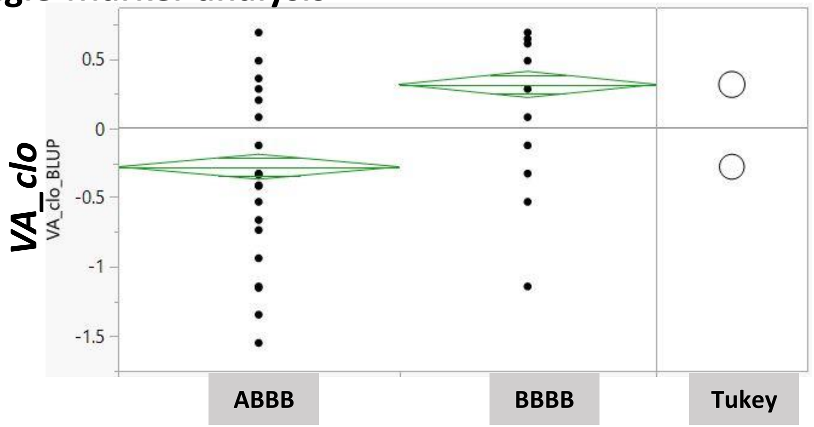

Genotypes	Freq.	Mean	Group
ABBB	96	-0.27691	X
BBBB	88	0.31612	Y

VA_clo_2019_ch10

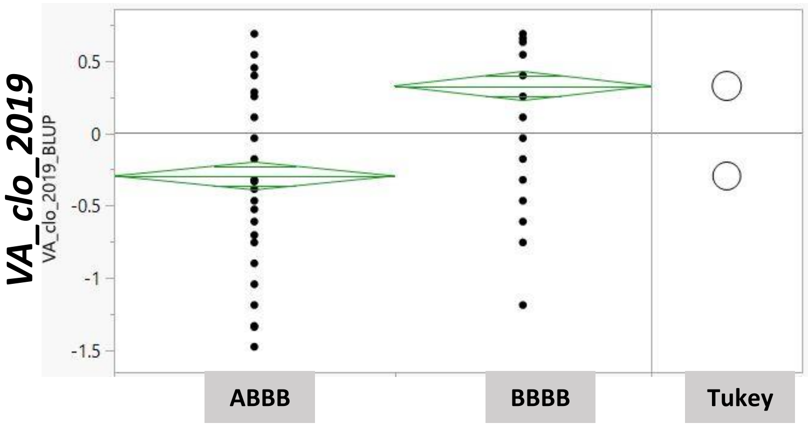

Genotypes	Freq.	Mean	Group
ABBB	96	-0.29262	X
BBBB	88	0.33098	Y

VA_clo_2020_ch10

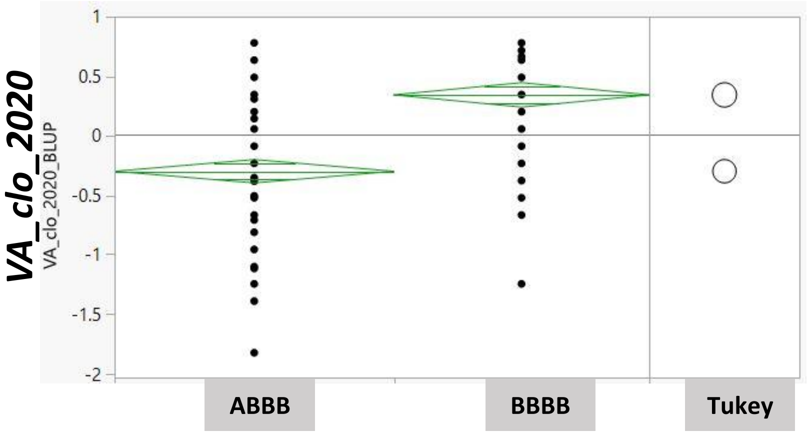

Genotypes	Freq.	Mean	Group
ABBB	96	-0.29799	X
BBBB	88	0.34327	Y

b_4

"solcap_snp_c1_8020" Single-Marker analysis

Mean comparison test (Tukey-Kramer; *p*-value < 0.05)

VA_clo_ch10

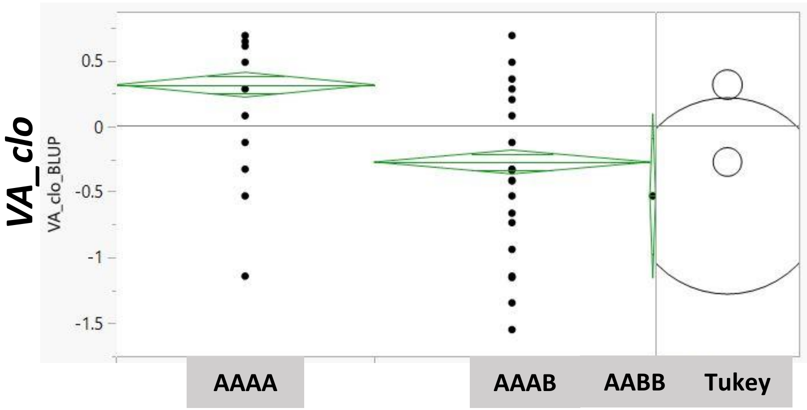

Genotypes	Freq.	Mean	Group
AAAA	88	0.31612	X
AAAB	94	-0.27153	Y
AABB	2	-0.5297	Y

VA_clo_2019_ch10

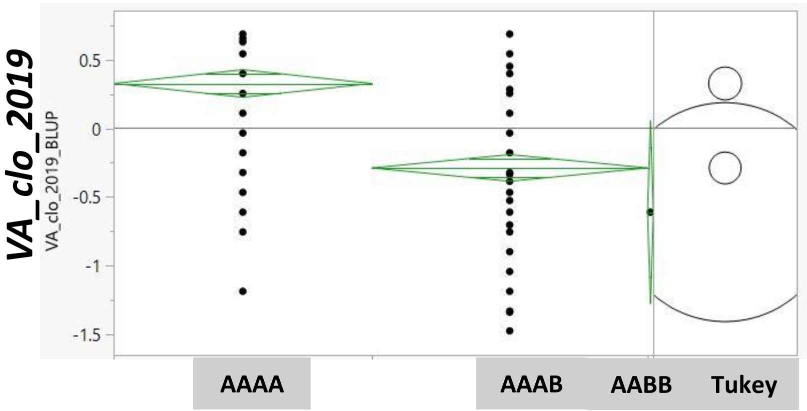

Genotypes	Freq.	Mean	Group
AAAA	88	0.33098	X
AAAB	94	-0.2859	Y
AABB	2	-0.60868	Y

VA_clo_2020_ch10

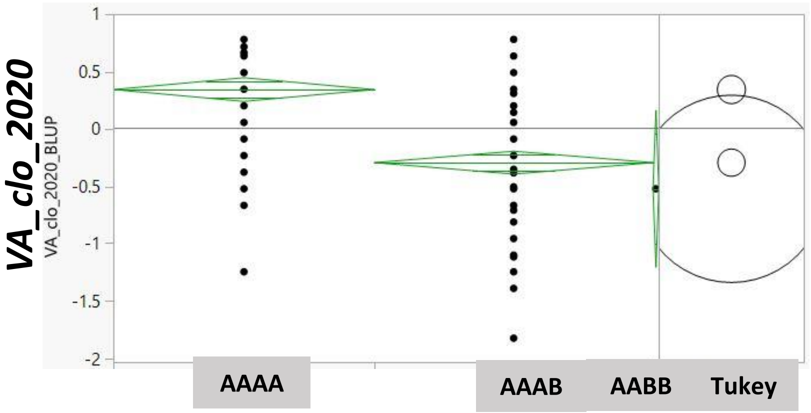

Genotypes	Freq.	Mean	Group
AAAA	88	0.34327	X
AAAB	94	-0.29325	Y
AABB	2	-0.5211	Y

b_5

"solcap_snp_c1_8019" Single-Marker analysis

Mean comparison test (Tukey-Kramer; *p*-value < 0.05)

VA_clo_ch10

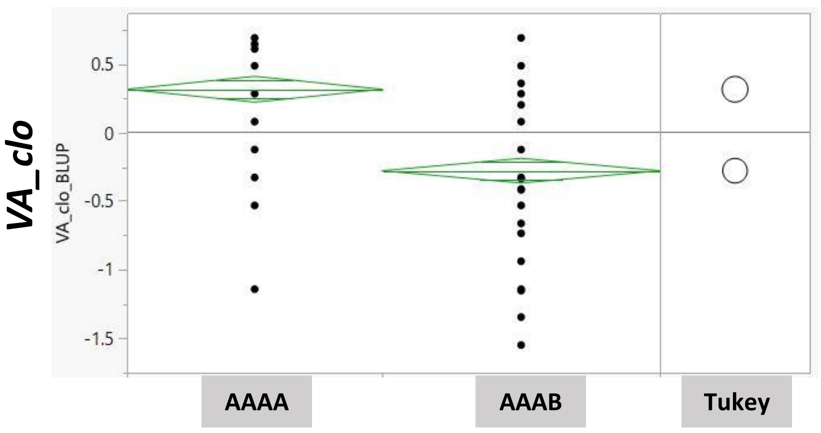

Genotypes	Freq.	Mean	Group
AAAA	88	0.31612	X
AAAB	96	-0.27691	Y

VA_clo_2019_ch10

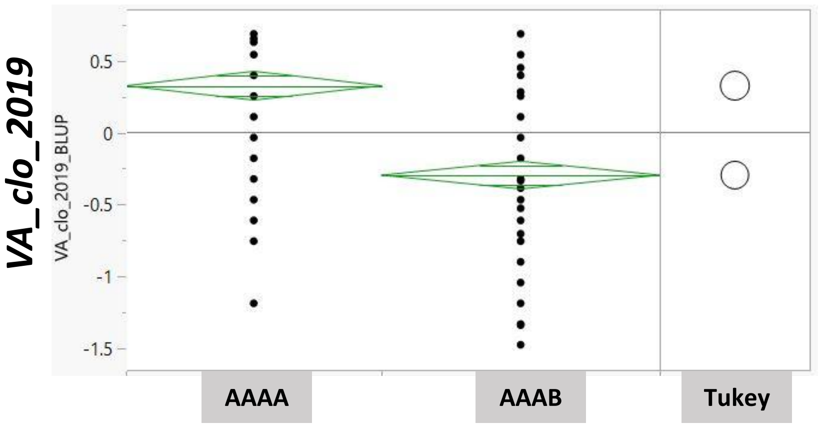

Genotypes	Freq.	Mean	Group
AAAA	88	0.33098	X
AAAB	96	-0.29262	Y

VA_clo_2020_ch10

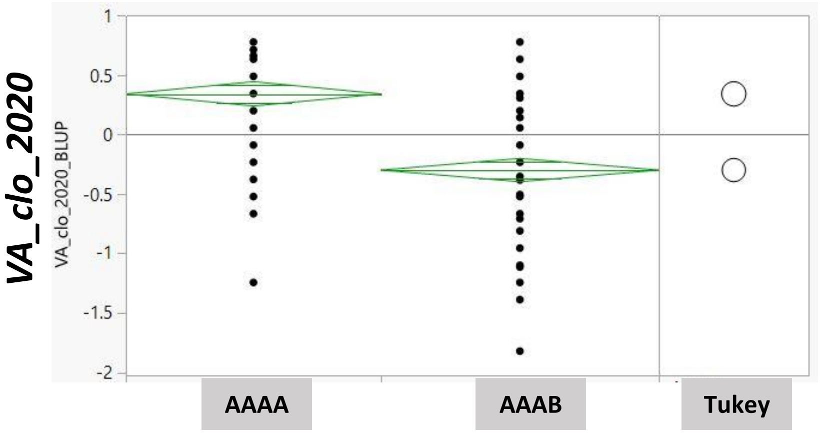

Genotypes	Freq.	Mean	Group
AAAA	88	0.34327	X
AAAB	96	-0.29799	Y

b_6

"PotVar0075244" Single-Marker analysis

Mean comparison test (Tukey-Kramer; *p*-value < 0.05)

VA_clo_ch04

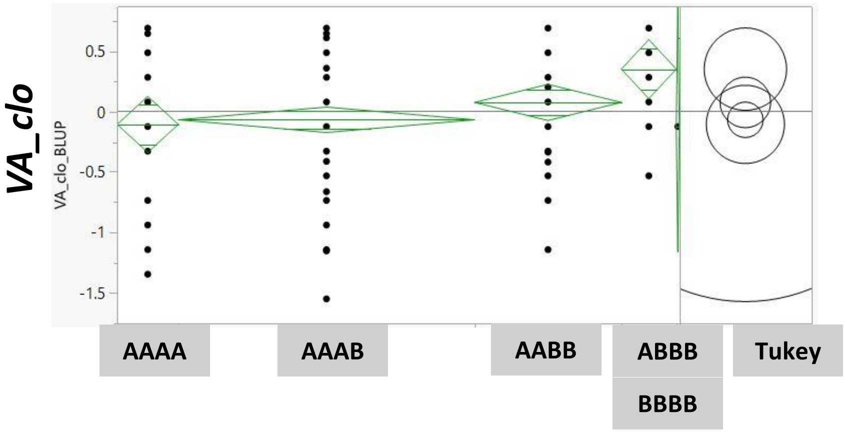

Genotypes	Freq.	Mean	Group
AAAA	20	-0.10507	XY
AAAB	97	-0.06731	X
AABB	48	0.07642	XY
ABBB	18	0.35118	Y
BBBB	1	-0.12314	XY

VA_clo_2019_ch04

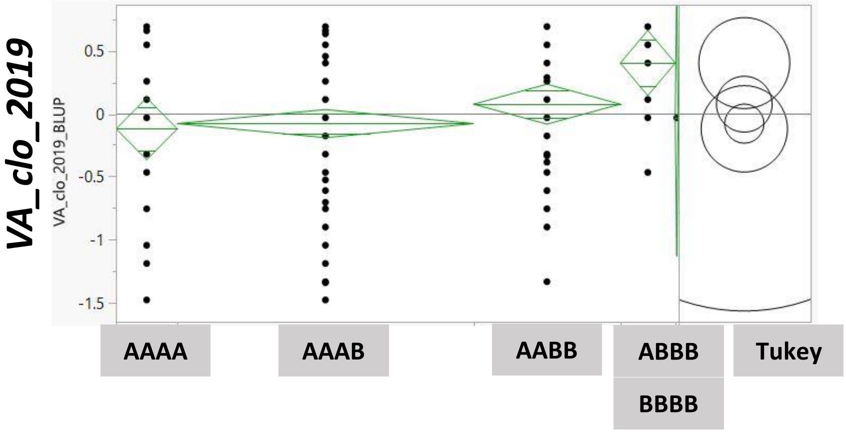

Genotypes	Freq.	Mean	Group
AAAA	20	-0.11891	X
AAAB	97	-0.07732	X
AABB	48	0.07685	XY
ABBB	18	0.40298	Y
BBBB	1	-0.03059	XY

VA_clo_2020_ch04

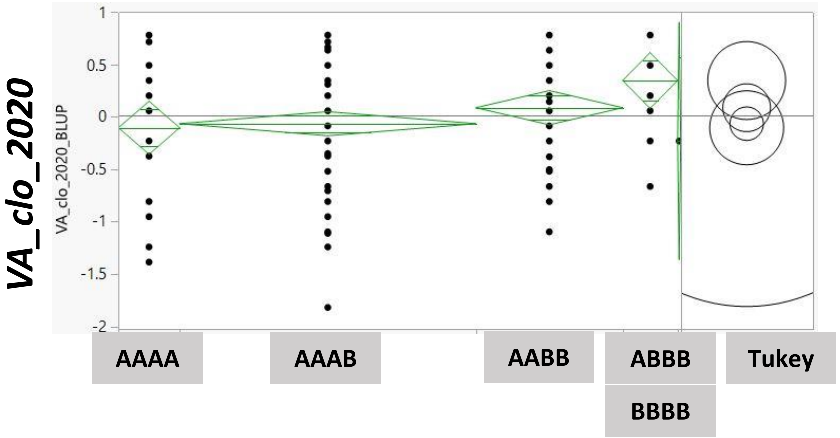

Genotypes	Freq.	Mean	Group
AAAA	20	-0.10519	XY
AAAB	97	-0.06625	X
AABB	48	0.08614	XY
ABBB	18	0.34604	Y
BBBB	1	-0.23205	XY

c

“solcap_snp_c2_41980” Single-Marker analysis

Mean comparison test (Tukey-Kramer; *p*-value < 0.05)

WD_clo_ch02

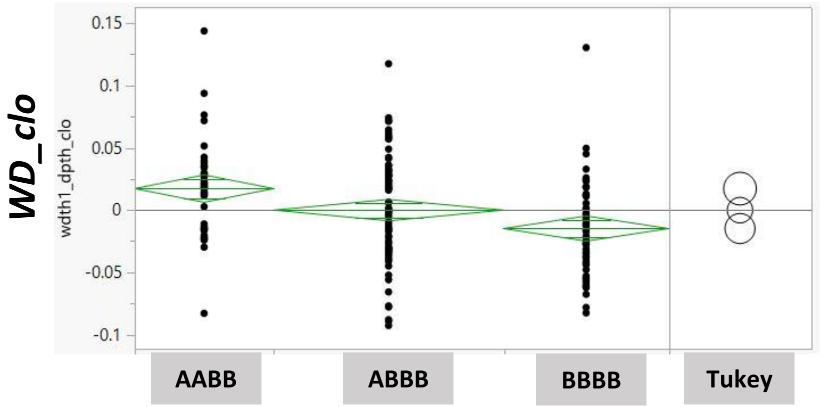

Genotypes	Freq.	Mean	Group
AABB	48	0.0173	X
ABBB	79	-0.00003	Y
BBBB	57	-0.01483	Y

WD_clo_2019_ch02

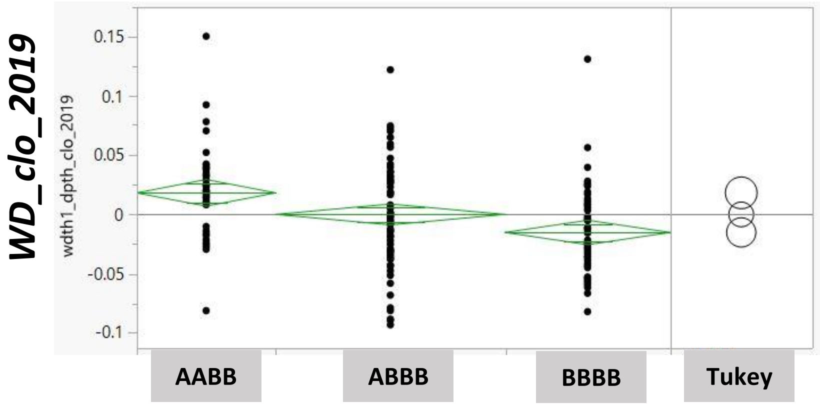

Genotypes	Freq.	Mean	Group
AABB	48	0.01811	X
ABBB	79	-0.0001	Y
BBBB	57	-0.0153	Y

WD_clo_2020_ch02

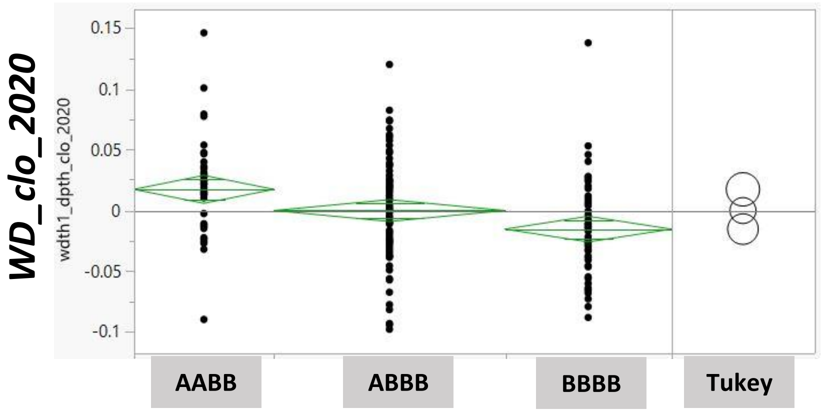

Genotypes	Freq.	Mean	Group
AABB	48	0.01748	X
ABBB	79	3.34E-05	XY
BBBB	57	-0.01522	Y

d_1

“solcap_snp_c1_10725” Single-Marker analysis

SG_clo_ch03

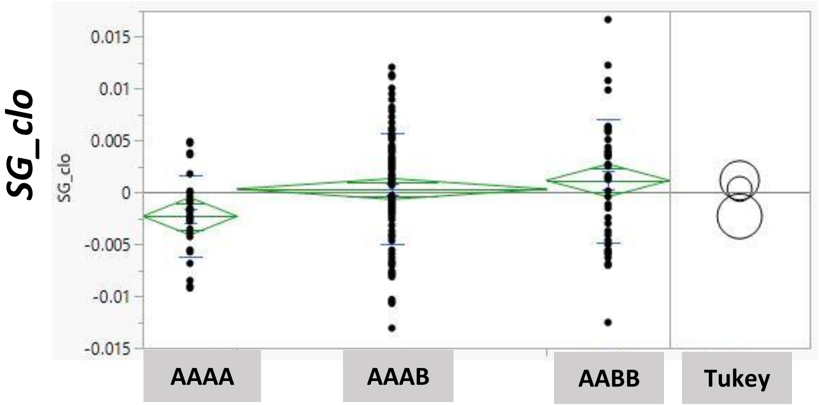

Mean comparison test (Tukey-Kramer; *p*-value < 0.05)

Genotypes	Freq.	Mean	Group
AAAA	33	-0.00229	X
AAAB	108	0.000339	Y
AABB	43	0.001163	Y

SG_clo_2020_ch03

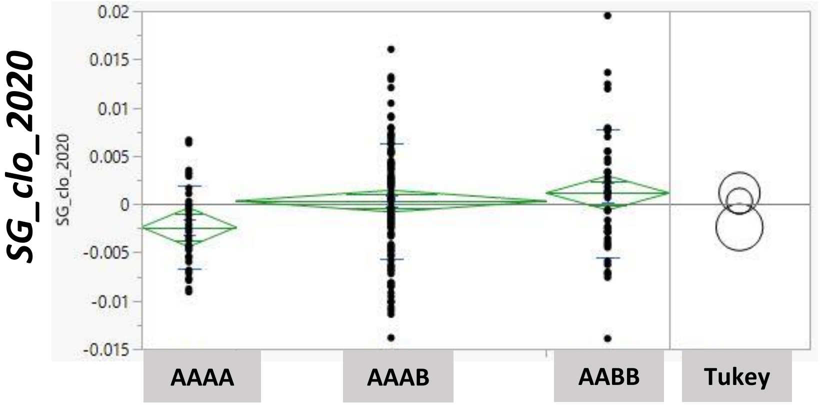

Genotypes	Freq.	Mean	Group
AAAA	33	-0.00239	X
AAAB	108	0.000315	XY
AABB	43	0.00116	Y

d_2

"PotVar0121927" Single-Marker analysis

Mean comparison test (Tukey-Kramer; *p*-value < 0.05)

SG_clo_ch03

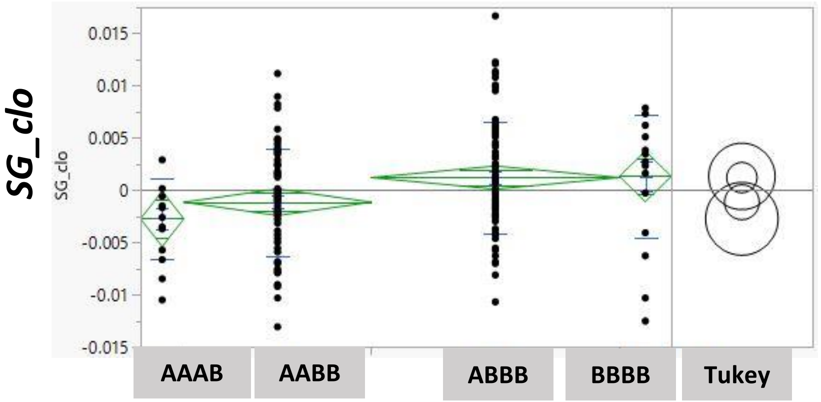

Genotypes	Freq.	Mean	Group
AAAB	15	-0.00274	X
AABB	65	-0.00115	X
ABBB	86	0.001203	Y
BBBB	18	0.001318	XY

SG_clo_2020_ch03

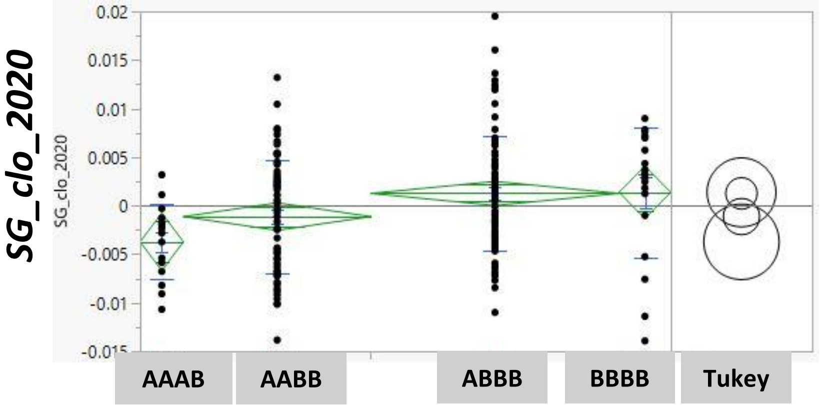

Genotypes	Freq.	Mean	Group
AAAB	15	-0.00372	X
AABB	65	-0.00112	XY
ABBB	86	0.001266	Y
BBBB	18	0.001379	XY
