# Supplementary material for: QTL identified that influence tuber length–width ratio, degree of flatness, tuber size, and specific gravity in a russet-skinned, tetraploid mapping population
Source: Front Plant Sci. 2024 Mar 22;15:1343632. doi: 10.3389/fpls.2024.1343632 (PMC10996053; doi:10.3389/fpls.2024.1343632)
Supplement: Supplementary file 9 [file Table_3.docx]

**Supplementary Table 3. Genome** **sequence coordinates and associated genes adjacent to pivotal SNPs**

| Significant QTL | Pivotal SNP linked to the significant QTL | Index | Genome coordinates found within the pivotal SNP's surrounding 200 kb interval. | Brief description of the gene obtained from PGSC v4.03 |
| --- | --- | --- | --- | --- |
| VA_clo_ch04 VA_clo_2019_ch04 VA_clo_2020_ch04 | PotVar0075244 | 1 | PGSC0003DMG400035662 | Gene of unknown function |
|  |  | 2 | PGSC0003DMG402007944 | Gene of unknown function |
|  |  | 3 | PGSC0003DMG401007944 | Gene of unknown function |
|  |  | 4 | PGSC0003DMG400008000 | L-asparaginase |
|  |  | 5 | PGSC0003DMG400008001 | D-type cyclin family 3 subgroup 3 |
|  |  | 6 | PGSC0003DMG400008002 | DCL protein |
|  |  | 7 | PGSC0003DMG400041336 | F-box family protein |
|  |  | 8 | PGSC0003DMG400007945 | Rer1 protein |
|  |  | 9 | PGSC0003DMG400007946 | Conserved gene of unknown function |
|  |  | 10 | PGSC0003DMG400008003 | Carbohydrate esterase |
|  |  | 11 | PGSC0003DMG400008035 | Gene of unknown function |
|  |  | 12 | PGSC0003DMG400008004 | Cysteine protease |
|  |  | 13 | PGSC0003DMG400007947 | WRKY transcription factor 2 |
|  |  | 14 | PGSC0003DMG400008036 | Serine-threonine protein kinase, plant-type |
|  |  | 15 | PGSC0003DMG400007948 | ATP binding protein |
|  |  | 16 | PGSC0003DMG400007949 | Conserved gene of unknown function |
|  |  | 17 | PGSC0003DMG400008006 | Kinesin heavy chain |
|  |  | 18 | PGSC0003DMG400008007 | Gene of unknown function |
|  |  | 19 | PGSC0003DMG400007950 | Ubiquitin carrier protein |
|  |  | 20 | PGSC0003DMG400008008 | Conserved gene of unknown function |
|  |  | 21 | PGSC0003DMG400007951 | DRE binding protein 1 |
|  |  |  |  |  |
| LW_clo_2020_ch06 | c2_31648 * | 1 | PGSC0003DMG400016276 | Acyltransferase |
|  |  | 2 | PGSC0003DMG400016275 | Acyltransferase |
|  |  | 3 | PGSC0003DMG400016347 | Gene of unknown function |
|  |  | 4 | PGSC0003DMG400041080 | ‘chromo’ domain containing protein |
|  |  | 5 | PGSC0003DMG400016274 | Protein kinase |
|  |  | 6 | PGSC0003DMG400016273 | Conserved gene of unknown function |
|  |  | 7 | PGSC0003DMG402016272 | Gene of unknown function |
|  |  | 8 | PGSC0003DMG401016272 | GTP binding protein gamma subunit |
|  |  | 9 | PGSC0003DMG400016314 | Conserved gene of unknown function |
|  |  | 10 | PGSC0003DMG400016313 | Calmodulin NtCaM13 |
|  |  | 11 | PGSC0003DMG400016271 | Gene of unknown function |
|  |  | 12 | PGSC0003DMG400016270 | Heat stress transcription factor A-6b |
|  |  | 13 | PGSC0003DMG400016268 | Chlorophyllase |
|  |  | 14 | PGSC0003DMG400016312 | Gene of unknown function |
|  |  | 15 | PGSC0003DMG400016346 | Gene of unknown function |
|  |  |  |  |  |
| WD_clo_ch02 WD_clo_2019_ch02 WD_clo_2020_ch02 | c2_41980 * | 1 | PGSC0003DMG400010430 | Conserved gene of unknown function |
|  |  | 2 | PGSC0003DMG400010451 | Gene of unknown function |
|  |  | 3 | PGSC0003DMG400010429 | Gene of unknown function |
|  |  | 4 | PGSC0003DMG400010444 | Conserved gene of unknown function |
|  |  | 5 | PGSC0003DMG400010443 | Conserved gene of unknown function |
|  |  | 6 | PGSC0003DMG400040656 | Gene of unknown function |
|  |  | 7 | PGSC0003DMG400010442 | Pentatricopeptide repeat-containing protein |
|  |  | 8 | PGSC0003DMG400010441 | Pyrroline-5-carboxylate reductase |
|  |  | 9 | PGSC0003DMG400010428 | TRNA (Guanine-n(7)-)-methyltransferase |
|  |  | 10 | PGSC0003DMG400010427 | Kinase |
|  |  | 11 | PGSC0003DMG400010440 | Ankyrin repeat-containing protein |
|  |  | 12 | PGSC0003DMG400010439 | CHP-rich zinc finger protein |
|  |  | 13 | PGSC0003DMG400010426 | Conserved gene of unknown fuction |
|  |  | 14 | PGSC0003DMG400010438 | ParB-like nuclease |
|  |  | 15 | PGSC0003DMG400041053 | Gene of unknown function |
|  |  | 16 | PGSC0003DMG400010424 | Rubisco subunit binding-protein alpha subunit, ruba |
|  |  | 17 | PGSC0003DMG401010423 | 2-oxo acid dehydrogenase, lipoyl-binding site |
|  |  | 18 | PGSC0003DMG402010423 | 2-oxo acid dehydrogenase, lipoyl-binding site |
|  |  | 19 | PGSC0003DMG400010450 | Glycoprotein endopeptidase |
|  |  | 20 | PGSC0003DMG400010422 | Pentatricopeptide repeat-containing protein |
|  |  | 21 | PGSC0003DMG400010437 | Structual constituent of ribosome |
|  |  |  |  |  |
|  |  |  |  |  |
| SG_clo_ch03 SG_clo_2020_ch03 | c1_3348 * | 1 | PGSC0003DMG400035756 | Gene of unknown function |
|  |  | 2 | PGSC0003DMG400035054 | Conserved gene of unknown function |
|  |  | 3 | PGSC0003DMG400013960 | Gene of unknown function |
|  |  | 4 | PGSC0003DMG400042551 | Gene of unknown function |
|  | c1_10725 * ^a^  c1_10734 * ^a^ | 1 | PGSC0003DMG400044643 | YALI0C15532p |
|  |  | 2 | PGSC0003DMG400040001 | Gene of unknown function |
|  |  | 3 | PGSC0003DMG400016917 | Conserved gene of unknown function |
|  |  | 4 | PGSC0003DMG400016922 | Glycine-rich RNA-binding protein |
|  |  | 5 | PGSC0003DMG400039520 | Gene of unknown function |
|  |  | 6 | PGSC0003DMG400016921 | Histone H2B |
|  |  | 7 | PGSC0003DMG400016920 | Splicing factor |
|  |  | 8 | PGSC0003DMG400016925 | Gene of unknown function |
|  |  | 9 | PGSC0003DMG400016924 | Gene of unknown function |
|  |  | 10 | PGSC0003DMG400016919 | Glu-rich protein |
|  | PotVar0121932 ^b^ PotVar0121927 ^b^ | 1 | PGSC0003DMG400040001 | Gene of unknown function |
|  |  | 2 | PGSC0003DMG400016917 | Conserved gene of unknown function |
|  |  | 3 | PGSC0003DMG400016922 | Glycine-rich RNA-binding protein |
|  |  | 4 | PGSC0003DMG400039520 | Gene of unknown function |
|  |  | 5 | PGSC0003DMG400016921 | Histone H2B |
|  |  | 6 | PGSC0003DMG400016920 | Splicing factor |
|  |  | 7 | PGSC0003DMG400016925 | Gene of unknown function |
|  |  | 8 | PGSC0003DMG400016924 | Gene of unknown function |
|  |  | 9 | PGSC0003DMG400016919 | Glu-rich protein |
|  |  | 10 | PGSC0003DMG400043666 | Gene of unknown function |
|  |  |  |  |  |
| TW_clo_2020_ch05 | c2_50176 * | 1 | PGSC0003DMG400021635 | Conserved gene of unknown function |
|  |  | 2 | PGSC0003DMG400021636 | Phosphomannomutase |
|  |  | 3 | PGSC0003DMG400021637 | Threonine aspartase |
|  |  | 4 | PGSC0003DMG400034667 | Conserved gene of unknown function |
|  |  | 5 | PGSC0003DMG400021638 | Conserved gene of unknown function |
|  |  | 6 | PGSC0003DMG400021652 | KDEL motif-containing protein 1 |
|  |  | 7 | PGSC0003DMG400021653 | RNA binding / adenosine deaminase |
|  |  | 8 | PGSC0003DMG400021639 | Remorin |
|  |  | 9 | PGSC0003DMG400021654 | R2r3-myb transcription factor |

^a^ The distance between the c1_10725 and c1_10734 was only 9,807 base pairs, so the genome coordinates found within those SNP's surrounding 200 kbp interval are the same.

^b^ The distance between the PotVar0121932 and PotVar0121927 was only 201 base pairs, so the genome coordinates found within those SNP's surrounding 200 kbp interval are the same.

* “solcap_snp_” was omitted at the beginning of all the SNP marker names beginning with either “c1” or “c2.”
